# Covered Self-Expandable Metallic Stents versus Multiple Plastic Stents for Benign Biliary Strictures: A Systematic Review, Meta-Analysis, and Trial Sequential Analysis

**DOI:** 10.1055/s-0046-1818623

**Published:** 2026-03-24

**Authors:** Tarek Nayfeh, Abdul Monaem Majzoub, Tarek Aboursheid, Alexander Malik, Karim Osman, Leslie Hassett, Jehan Mousa, Konstantinos Malandris, Nabil Noureddin, Azizullah Ahmadallah Beran, Faisal Kamal, Nasir Saleem

**Affiliations:** 1 Department of Medicine, MedStar Health Georgetown University (Baltimore) Internal Medicine Residency Program, Baltimore, Maryland, United States; 2 Division of Gastroenterology and Hepatology, Department of Medicine, University of Missouri School of Medicine, Columbia, Missouri, United States; 3 Department of Internal Medicine, Ascension Saint Francis Hospital, Evanston, Illinois, United States; 4 Division of Gastroenterology and Hepatology, Department of Medicine, MetroHealth, Cleveland, Ohio, United States; 5 Division of Gastroenterology and Hepatology, Department of Medicine, Lahey Hospital and Medical Center, Burlington, Massachusetts, United States; 6 Mayo Clinic Libraries, Mayo Clinic, Rochester, Minnesota, United States; 7 Clinical Research and Evidence-Based Medicine Unit, Second Medical Department, Aristotle University of Thessaloniki, Thessaloniki, Greece; 8 Division of Gastroenterology and Hepatology, Department of Medicine, University of California San Diego School of Medicine, California, United States; 9 Division of Gastroenterology and Hepatology, Department of Medicine, Indiana University School of Medicine, Indianapolis, Indiana, United States; 10 Division of Gastroenterology and Hepatology, Department of Medicine, Thomas Jefferson University Hospital, Philadelphia, Pennsylvania, United States

**Keywords:** benign, biliary, stricture, stent, plastic, metallic, meta-analysis

## Abstract

**Purpose:**

We aim to compare the benefits, harms, and costs of multiple plastic stents (MPS) versus covered self-expandable metallic stents (CSEMS) in the treatment of benign biliary strictures (BBS).

**Methods:**

A comprehensive database search through June 6, 2024 identified randomized controlled trials (RCT) involving adult patients with BBS comparing MPS to CSEMS in terms of stricture resolution, recurrence, technical success, number of required procedures and stents, treatment duration, all-cause mortality, adverse events, and stent migration. We utilized the Cochrane Risk of Bias (RoB) tool2 for study quality assessment. Meta-analysis following the random effects model was performed.

**Results:**

After screening 365 references, we included 7 RCTs (high RoB) involving 594 patients. Most reported stricture etiology is chronic pancreatitis (52.4%), followed by orthotopic liver transplant (40.5%). Compared with MPS, CSEMS achieved similar efficacy and safety. However, CSEMS required fewer procedures (mean difference [MD]: −1.84 [−2.56 to −1.11]; I ^2^ = 76%) and stents (MD: −6.04 [−9.55 to −2.52]; I ^2^ = 93%), amounting for shorter duration of treatment (MD: −119.02 days [−137.41 to −100.62]; I ^2^ = 96%) and savings in costs between 41 and 57% with absolute savings of 4,019$ to 9,192$ favoring CSEMS. All outcomes had a low to very low certainty of evidence (CoE), except for technical success and stricture resolution (moderate CoE).

**Conclusion:**

Our analysis suggests that CSEMSs are comparable to MPSs in terms of efficacy and safety. However, it shows that CSEMSs are more cost-effective. Sample size calculations from our analyses can guide the design of future studies to validate the safety profiles and determine the feasibility of the two modalities.

## Introduction

Biliary duct strictures can be due to congenital or acquired etiologies. Acquired etiologies are far more common and can be further classified into malignant and benign causes. Malignancies contribute to almost 70% of the cases and are mostly due to pancreatic head cancer and cholangiocarcinoma. Benign etiologies constitute the remaining 30% of biliary strictures, 1 most commonly due to biliary duct injury post-cholecystectomy, post-orthotopic liver transplantation (OLT), and chronic pancreatitis (CP). Other etiologies are less common. 1 2 3 4 Supplementary Table S1 (available in the online version only) shows benign and malignant etiologies of biliary duct strictures.

The clinical presentation of biliary strictures ranges from asymptomatic abnormal liver enzymes to symptoms associated with stasis, including jaundice and pruritus. If left untreated, biliary strictures can lead to more severe conditions, including cholangitis, cirrhosis, and hepatic failure. 1

Treatment of biliary duct stricture aims at restoring normal biliary flow and relieving obstructive symptoms. Approaches include surgery, percutaneous hepatic intervention, and endoscopic management. Endoscopic stenting stands as a more favorable approach, given that it is less invasive and has comparable efficacy of 76 to 84%. Biliary stents can be either plastic or metallic. Multiple limitations accompany the use of plastic stents, including rigidity, the need for multiple endoscopic retrograde cholangiopancreatography (ERCP) sessions, and the necessity of frequent stent exchange to achieve long-term resolution, which drives up the costs, reduces patient compliance, and is associated with morbidity. 5 However, metallic stents only require two ERCP sessions, one for deployment and one for retrieval, or, in the case of spontaneous passage of the stent, even one ERCP procedure is sufficient. 6

The current American College of Gastroenterology (ACG) guideline recommends fully covered self-expandable metallic stents (CSEMS) over multiple plastic stents (MPS) for treating extrahepatic biliary strictures due to convenience. However, this recommendation is conditional and is based on low-quality evidence from observational studies. 4 The optimal endoscopic management of benign biliary strictures (BBS) remains debated, with the choice of stent largely dependent on the endoscopist's discretion. In this study, we conducted a systematic review of the literature to compare the efficacy, safety, and costs of MPS and CSEMS in the treatment of BBS.

## Methods

This systematic review and meta-analysis was performed in accordance with the Preferred Reporting Items for Systematic Reviews and Meta-Analyses (PRISMA) guidelines. 7 This systematic review was exempted from institutional review board approval as it does not involve any interaction or intervention with human subjects, nor does it include access to identifiable private information.

### Eligibility Criteria

We included randomized controlled trials (RCTs) involving adults (aged 18 years or older), with only BBS, comparing fully or partially CSEMS to MPS. Our primary outcome is stricture resolution, defined as the absence of remaining lesion detected through radiological evaluation, normal liver function tests, absence of symptoms of nonblocked cholangitis during follow-up, no requirement for additional stenting, or any combination of these factors. Secondary outcomes include stricture resolution after initial treatment; technical success, defined as successful placement and/or removal of stents; stricture recurrence; number of ERCP procedures; number of stents; stent treatment duration in days; all-cause mortality; adverse events; and stent migration.

### Information Sources

A comprehensive search of several databases was performed on June 6, 2024. Results were limited to the English Language. No date limits for the search were applied. Databases searched (and their content coverage dates) were Ovid MEDLINE(R) (1946+ including epub ahead of print, in-process, and other nonindexed citations), Ovid Embase (1974 + ), Ovid Cochrane Central Register of Controlled Trials (1991 + ), Ovid Cochrane Database of Systematic Reviews (2005 + ), and Scopus via Elsevier (1970 + ).

The search strategies were designed and conducted by a medical librarian with input from the study investigators. Controlled vocabulary supplemented with keywords was used. The actual strategies listing all search terms used and how they are combined are available in the Supplementary Materials , available in the online version only. 8

### Selection Process

References retrieved from the literature search were stored and managed in an EndNote library and then uploaded to Covidence, a web-based collaboration software platform that streamlines the production of systematic and other literature reviews. For abstract screening, two human reviewers screened each abstract independently. Consensus for inclusion and any abstracts with conflicting recommendations were advanced for full-text screening. Independent reviewers, working in pairs, screened the full-text version of eligible references. Discrepancies between the reviewers were resolved through discussions and consensus. When consensus could not be reached, a third reviewer resolved the difference.

### Data Collection Process and Data Items

Two human reviewers independently extracted the data. Discrepancies between the reviewers were resolved through discussions and consensus. We used piloted extraction forms on Excel, which include the following items for each study: author, year, country, study period, follow-up, trial registration number, funding, inclusion and exclusion criteria, details of intervention, total number randomized, age, sex, stricture etiology, time to stricture development from time of insult, bilirubin and alkaline phosphatase, primary and secondary outcomes, unit of measurement, and data analysis (per-protocol and intention-to-treat).

### Study Risk of Bias Assessment

Two human reviewers assessed the risk of bias of the included studies independently. Discrepancies between the reviewers were resolved through discussions and consensus. We used the Cochrane RoB2 tool to assess the risk of bias of the included RCTs arising from five domains: randomization, deviations from intended interventions, missing outcome data, measurement of outcome, and selection of the reported result. 9

### Effect Measures and Synthesis Methods

For dichotomous variables, we calculated the risk ratio (RR) and 95% confidence interval (CI). For adverse events and stent migration, we calculated the incidence rate ratio (IRR) and 95% CI. For continuous data, we calculated the mean difference (MD) and 95% CI between treatment groups. We converted medians and ranges into means and standard deviations following the methods suggested by Hozo et al. 10 When possible, we performed meta-analyses following the restricted maximum-likelihood random-effects model in an intention-to-treat approach.

We conducted subgroup analyses based on the etiology of BBS. We used the Chi-square test to test for subgroup interactions to examine differences in subgroups. We used the I ^2^ statistic to quantify the variability in effect estimates that is due to genuine subgroup differences rather than sampling error. 11

For sensitivity analyses, we repeated the analyses following the fixed-effects model, assessed imprecision with trial sequential analyses (TSAs), and for the primary outcome, we performed sensitivity analyses in a per-protocol approach and in an intention-to-treat approach, where we imputed missing data according to the following two extreme scenarios:

- “Best case scenario” favoring the CSEMS group: all the dropouts/participants lost to follow-up in the CSEMS group—but none of the dropouts/participants lost to follow-up in the MPS group—were assumed to have experienced the outcome, including all randomized participants in the denominator.
- “Worst case scenario” favoring the MPS group: all the dropouts/participants lost to follow-up in the MPS group—but none of the dropouts/participants lost to follow-up in the CSEMS group—were assumed to have experienced the outcome, including all randomized participants in the denominator.

Heterogeneity was assessed by visual inspection of the forest plots, using a standard Cochrane Chi-squared test (significance level at 0.1), and quantified using the I ^2^ statistic (I ^2^ ≥ 50% indicates substantial heterogeneity). 11

### Publication Bias Assessment

We could not assess for publication bias as the number of included trials is less than 10 and as such the power of the tests is low per the recommendations of the Cochrane Handbook for Systematic Reviews of Interventions. 12

### Trial Sequential Analysis

We performed TSAs due to the risk of random errors in cumulative meta-analyses, which can arise from sparse data and repeated testing of accumulating data. To manage these errors, we computed the required information size. This calculation also factors in the diversity present in the meta-analysis. We used diversity as the I ^2^ statistic may underestimate the required information size when adjusting for heterogeneity. 13 14 15

For dichotomous outcomes, we based the diversity-adjusted required information size (DARIS) on the event proportion in the control group, an assumed a priori relative risk reduction of 25% (except for stricture resolution, relative risk reduction of 20% corresponding with a risk difference of 15%), a risk of type I error of 0.9% (by dividing the conventional threshold of 5% by 5.5—a number midway between 1 and total number of outcomes, which is 10), 16 a risk of type II error of 10%, and the observed diversity of the included trials in the meta-analysis. 17 For the continuous outcomes, we estimated the required information size based on the variance estimated from the pooled results and the observed diversity in the trials in the meta-analysis. We also calculated and reported the TSA-adjusted CI. 18

The underlying assumption of TSA is that testing for significance may be performed each time a new trial is added to the meta-analysis. We added the trials according to the year of publication, and, if more than one trial has been published in a year, trials were added alphabetically according to the last name of the first author.

Based on DARIS, we constructed trial sequential monitoring boundaries. These boundaries determine the statistical inference that can be drawn about the cumulative meta-analysis that has not reached the required information size. If the cumulative Z-curve crosses the trial sequential monitoring boundary for benefit or harm before the DARIS is reached, we may have established firm evidence. Similarly, if the cumulative Z-curve crosses the monitoring boundary for futility before DARIS is reached, we may reach the conclusion that the two interventions do not differ, and in either case, further trials may be unnecessary. We performed TSA with Trial Sequential Analysis software, version 0.9.5.10 β (TSA 2017). 18

### Certainty Assessment

We created a summary-of-findings table for the primary and secondary outcomes. We followed the Grading of Recommendations, Assessment, Development, and Evaluations (GRADE) approach in the assessment of the certainty of evidence (CoE). RCTs' CoE assessment starts at high and is rated down for one or more of the following five domains: study limitations (rated down by one level for major concerns in the risk of bias), indirectness, inconsistency (rated down by one level for substantial heterogeneity I ^2^ ≥ 50%, and by two levels for severe heterogeneity I ^2^ ≥ 90%), imprecision (we followed a minimally contextualized approach in which we considered an arbitrary threshold of 15% risk difference between CSEMS and MPS to be minimally clinically important for the primary outcome, based on the judgment of five board-certified gastroenterologists who are experts in ERCP. 19 For the secondary outcomes, we considered an arbitrary threshold at which the sample size reaches an optimal information size required to achieve a 90% chance of detecting, at a significance level of 0.9%, a risk difference between CSEMS and MPS corresponding to a relative risk difference of 25%. For continuous outcomes, we considered an optimal information size required to achieve a 90% chance of detecting, at a significance level of 0.9%, a difference corresponding to the variance estimated from the pooled results. We rated down by one level for imprecision if the CI overlaps the threshold of interest. If the CI overlaps the threshold and one or both boundaries suggest different inferences from the point estimate, we rated down by two levels. For a considerably wide CI where the two boundaries indicate very different inferences, we rated down by three levels. In TSA, where the cumulative Z-value does not cross the monitoring boundaries for benefit, harm, or futility, we rated down our assessment by two levels if the accrued number of participants is below 50% of the DARIS, and by one level if between 50 and 100% of DARIS. We did not rate down for imprecision if the cumulative Z-value reaches or crosses benefit, harm, futility, or DARIS), and publication bias. 20 21 22 23 24 25 26

## Results

### Study Selection and Study Characteristics

**Fig. 1:**
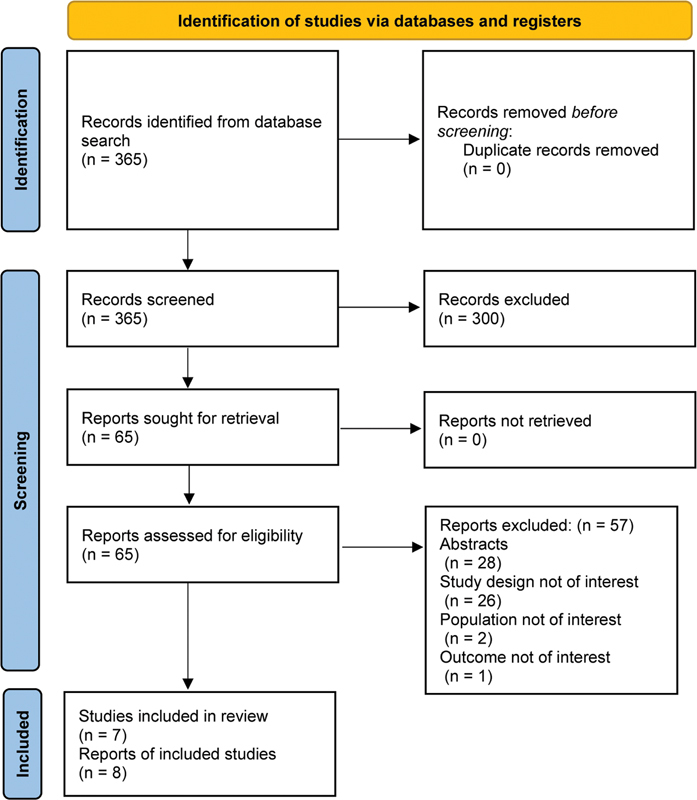
PRISMA flow diagram illustrating study identification and selection process.

Our search of the literature yielded 365 unique references. After screening the titles and abstracts, we included 65 references for further review. Following full-text screening, we included eight reports that involved seven RCTs in our systematic review. 19 27 28 29 30 31 32 33 Two references reported on the same RCT. 28 33 The PRISMA flow diagram shows the study selection process ( Fig. 1 ).

The 7 RCTs involved a total of 594 patients. In addition, 246 patients were in the CSEMS group (median age range: 45.5–57 years; 27.2% females) and 248 patients were in the MPS group (median age range: 45.2–58.5 years; 24.2% females). Most reported stricture etiology is CP (52.4%), followed by OLT (40.5%) and post-surgical injury (7.1%), mainly due to cholecystectomy (5.5%). Median time to stricture development from the index insult ranged between 1 and 37 months. Median follow-up time ranged between 12 and 72 months. Full description of eligibility criteria, details of stenting, and baseline characteristics of the included RCTs are available in Table 1 and Supplementary Table S2 (available in the online version only). 8

**Table 1:** Baseline characteristics

Author, year; country; study period; clinical trial registration number	Intervention (No. of patients)	Age (years) a	Female, *n* (%)	Stricture etiology, *n* (%)	Time to stricture development (months) a	ALP (IU/L); BR (mg/dL) a	Follow-up (months) b
Artifon, 2012 27 ; Brazil; 2002 to 2006; NR	PCSEMS (15)	Mean 45.5	10 (67)	Post-surgical strictures 31 (100): cholecystectomy 27 (87), partial hepatectomy 1 (3), bariatric gastroplasty 1 (3), total gastrectomy 1 (3), and fundoplication 1 (3)	1.16	719 ± 205; 9.44 ± 2.15	60–72
	MPS (16)	Mean 45.2	10 (63)		1.02	779 ± 211; 9.18 ± 2.42	
Cantù, 2021 28 ; Finland, Germany, and Italy; 2012–2015; NCT01393067	FCSEMS (15)	59 (50–67)	3 (21)	Anastomotic strictures post-OLT 30 (100)	7 (1–58)	260 ± 146; 2 ± 2	63 (41–80)
	MPS (15)	55 (22–68)	1 (7)		6 (2–89)	254 ± 145; 4 ± 3	55 (34–74)
Coté, 2016 19 ; United States and United Kingdom; 2011–2014; NCT01221311	CSEMS (57)	54.5 ± 10.4	19 (33)	Anastomotic strictures post-OLT 37 (65), CP 18 (32), other postoperative injury 2 (3)	3 (1–44)	240 (14–1,615); 1.4 (0.4–17.4)	12
	MPS (55)	56.7 ± 11	17 (31)	Anastomotic strictures post-OLT 36 (65), CP 17 (31), other postoperative injury 2 (4)	4 (1–96)	317 (52–1,027); 1.2 (0.4–20.7)	
Haapamäki, 2015 29 ; Finland; 2008–2012; NCT01085747	CSEMS (30)	54.5 (30–78)	5 (17)	CP 60 (100)	NR	487 (86–1,767); 4.2 (0.3–16.1)	41 (1–66)
	MPS (30)	49.5 (30–69)	1 (3)		NR	328 (66–1,040); 1.8 (0.4–14.3)	37 (3–61)
Kaffes, 2014 30 ; Australia; 2008–2011; NR	FCSEMS (10)	49.5 (23–69)	5 (50)	Anastomotic strictures post-OLT 20 (100)	26 (1–204)	NR	25.5 (3–44)
	MPS (10)	56.5 (38–67)	5 (50)		11.8 (1–30)	NR	26 (6–40)
Martins, 2018 31 ; Brazil; 2009–2014; NCT01148199	CSEMS (30)	54 (23–73)	8 (27)	Anastomotic strictures post-OLT 59 (100)	7.7 ± 9.2	NR	33 (3–64)
	MPS (29)	50 (28–71)	9 (31)		9.3 ± 15.4	NR	36 (0–77)
Ramchandani, 2021 32 ; International (11 countries); 2012–2020; NCT01543256	FCSEMS (80)	51 (28–74)	10 (13)	CP 164 (100)	37.2 (1.2–429.6)	337 (46–1,871); 1 (0.1,25.3)	Median 23.7 IQR (22.8–24.4)
	MPS (84)	53 (26–74)	12 (14)		20.4 (1.2–388.8)	262.5 (55–1,909); 1.4 (0.2,17.7)	
Tal, 2017 33 ; Finland, Germany and Italy; 2012–2015; NCT01393067	CSEMS (24)	57 (32–69)	10 (42)	Anastomotic strictures post-OLT 48 (100)	5.4 (0.3–259.3)	NR	14 (2–44)
	MPS (24)	58.5 (32–72)	6 (25)		13.9 (1.6–43.9)	NR	17 (2–39)

Abbreviations: ALP, alkaline phosphatase; BR, bilirubin; CP, chronic pancreatitis; CSEMS, covered self-expandable metal stents; FCSEMS, fully covered self-expandable metal stents; MPS, multiple plastic stents; NR, not reported; OLT, orthotopic liver transplant; PCSEMS, partially covered self-expandable metal stents.

a Reported as mean ± SD or median (range).

b Reported as median (range).

### Risk of Bias in Studies

All RCTs were at an overall high risk of bias due to high risk of bias in the deviations from intended intervention domain. 19 27 28 29 30 31 32 33 Lack of blinding of participants and personnel and lack of blinding of the endoscopist may have an impact on our outcomes of interest depending on the experience, expertise, and preference of the endoscopist performing the procedure. Some concerns were appreciated in the selection of the reported results (6 RCTs), 27 28 29 30 31 32 33 measurement of the outcome (3 RCTs), 30 31 32 missing outcome data (2 RCTs), 29 32 and randomization process domains (1 RCT; Supplementary Figs. S1 and S2 , available in the online version only). 8 33

### Results of Individual Studies, Meta-analyses, and CoE

Results of individual studies and all meta-analyses, including overall, subgroup, and sensitivity analyses, are shown in Supplementary Tables S3–S5 and Supplementary Figs. S3–S27 (available in the online version only). 8 A summary-of-findings table with full assessment of the CoE of all primary and secondary outcomes, with explanations of the assessments, can be found in Table 2 .

**Table 2:** Certainty of evidence assessment following GRADE

Outcomes	No. of participants (studies) Follow-up	Certainty of the evidence (GRADE)	Relative effect (95% CI)	Anticipated absolute effects	
				Risk with MPS	Risk difference with CSEMS
Stricture resolution	499 (7 RCTs)	⨁⨁⨁◯ Moderate a b c	RR 1.04 (0.97–1.11)	749 per 1,000	30 more per 1,000 (22 fewer to 82 more)
Stricture resolution after initial treatment	50 (2 RCTs)	⨁◯◯◯ Very low a c d e	RR 0.84 (0.33–2.15)	720 per 1,000	115 fewer per 1,000 (482 fewer to 828 more)
Stricture recurrence	251 (5 RCTs)	⨁◯◯◯ Very low a c f	RR 1.27 (0.66–2.45)	107 per 1,000	29 more per 1,000 (36 fewer to 155 more)
Technical success	307 (3 RCTs)	⨁⨁⨁◯ Moderate a c g	RR 1.00 (0.98–1.03)	987 per 1,000	0 fewer per 1,000 (20 fewer to 30 more)
Stent migration	499 (7 RCTs)	⨁◯◯◯ Very low a c h	IRR 1.13 (0.72–1.78)	143 per 1,000	19 more per 1,000 (40 fewer to 112 more)
All-cause mortality	501 (7 RCTs)	⨁◯◯◯ Very low a c i	RR 1.22 (0.41–3.62)	48 per 1,000	10 more per 1,000 (28 fewer to 125 more)
Adverse events	499 (7 RCTs)	⨁◯◯◯ Very low a c j	IRR 1.13 (0.86–1.47)	418 per 1,000	54 more per 1,000 (59 fewer to 197 more)
Stent treatment duration (days)	382 (5 RCTs)	⨁◯◯◯ Very low a c k l	–	The mean stent treatment duration (days) was 293.34 days	MD 119.02 days fewer (137.41 fewer to 100.62 fewer)
Number of ERCP procedures	382 (5 RCTs)	⨁⨁◯◯ Low a c m n	–	The mean number of ERCP procedures was 4.36 procedures	MD 1.84 procedures fewer (2.56 fewer to 1.11 fewer)
Number of stents	492 (7 RCTs)	⨁◯◯◯ Very low a c o p	–	The mean number of stents was 7.25 stents	MD 6.04 stents fewer (9.55 fewer to 2.52 fewer)

Abbreviations: CI, confidence interval; ERCP, endoscopic retrograde cholangiopancreatography; IRR, incidence rate ratio; CSEMS, covered self-expandable metal stents; MD, mean difference; MPS, multiple plastic stent; RCT, randomized controlled trial; RR, risk ratio.

Note: The risk in the intervention group (and its 95% confidence interval) is based on the assumed risk in the comparison group and the relative effect of the intervention (and its 95% CI).

GRADE Working Group grades of evidence:

• High certainty: we are very confident that the true effect lies close to that of the estimate of the effect.

• Moderate certainty: we are moderately confident in the effect estimate; the true effect is likely to be close to the estimate of the effect, but there is a possibility that it is substantially different.

• Low certainty: our confidence in the effect estimate is limited; the true effect may be substantially different from the estimate of the effect.

• Very low certainty: we have very little confidence in the effect estimate; the true effect is likely to be substantially different from the estimate of the effect.

a CoE was downgraded by one level due to serious study limitations. All studies were judged to be at high risk of bias for blinding of participants and personnel. Lack of blinding of the endoscopist may have an impact on stricture resolution, technical success, stricture recurrence, stent treatment duration, number of ERCP procedures, number of stents, or complications (stent migration, adverse events, all-cause mortality) depending on the experience, expertise, and preference of the endoscopist performing the procedure.

b CoE was not downgraded for imprecision: effect size suggests trivial benefit, the 95% CI does not overlap the arbitrary threshold of 15%, and the Z-curve crosses futility boundary even though DARIS is not reached (499 vs. 537 required to have a 90% chance of detecting, as significant at the 0.9% level, a 15% RD in stricture resolution between CSEMS and MPS.

c Publication bias was not assessed due to the small number of studies.

d CoE was downgraded by one level for inconsistency: I ^2^ = 75%.

e CoE was downgraded by three levels for imprecision: effect size suggests trivial harm with a possibility of very important benefit and harm as the 95% CI overlaps the arbitrary threshold of 18% (corresponding to 25% RRR), and the Z-curve did not cross benefit, harm, or futility boundaries and the sample size is <50% of the DARIS (50 vs. 1,220) required to have a 90% chance of detecting, as significant at the 0.9% level, an 18% RD in stricture resolution after initial treatment between CSEMS and MPS.

f CoE was downgraded by two levels for imprecision: effect size suggests important harm with a possibility of important benefit as the 95% CI overlaps the arbitrary threshold of 2.7% (corresponding to 25% RRR), and the Z-curve did not cross benefit, harm, or futility boundaries and the sample size is <50% of the DARIS (251 vs. 13,124) required to have a 90% chance of detecting, as significant at the 0.9% level, a 2.7% RD in stricture recurrence between CSEMS and MPS.

g CoE was not downgraded for imprecision: effect size suggests trivial to no effect, the 95% CI does not overlap the arbitrary threshold of 24.7% (corresponding to 25% RRR), although the Z-curve did not cross benefit, harm, or futility boundaries and the sample size is <50% of the DARIS (307 vs. 2,928) required to have a 90% chance of detecting, as significant at the 0.9% level, a 25% RD in technical success between CSEMS and MPS.

h CoE was downgraded by two levels for imprecision: effect size suggests trivial harm with a possibility of important benefit and harm as the 95% CI overlaps the arbitrary threshold of 3.6% (corresponding to 25% RRR), and the Z-curve did not cross benefit, harm, or futility boundaries and the sample size is <50% of the DARIS (499 vs. 8,285) required to have a 90% chance of detecting, as significant at the 0.9% level, a 3.6% RD in stent migration between CSEMS and MPS.

i CoE was downgraded by three levels for imprecision: effect size suggests trivial harm with a possibility of very important benefit and harm as the 95% CI overlaps the arbitrary threshold of 1.2% (corresponding to 25% RRR), and the Z-curve did not cross benefit, harm, or futility boundaries and the sample size is <50% of the DARIS (501 vs. 16,945) required to have an 90% chance of detecting, as significant at the 0.9% level, a 1.2% RD in all-cause mortality between CSEMS and MPS.

j CoE was downgraded by two levels for imprecision: effect size suggests trivial harm with a possibility of important harm as the 95% CI overlaps the arbitrary threshold of 10.5% (corresponding to 25% RRR), and the Z-curve did not cross benefit, harm, or futility boundaries and the sample size is <50% of the DARIS (499 vs 2,382) required to have an 90% chance of detecting, as significant at the 0.9% level, a 10.5% RD in adverse events between CSEMS and MPS.

k CoE was downgraded by two levels for Inconsistency: I ^2^ = 95%.

l CoE was not downgraded for imprecision: Z-curve crossed the benefit boundary and sample size reached DARIS (382 vs. 300). CoE downgrading was driven by the severe heterogeneity as the results of the random-effect model were imprecise (MD −98.62 [−180.80, −16.43]) compared with the common effects model (MD −119.02 [−137.41, −100.62]).

m CoE downgraded by one level for inconsistency: I ^2^ = 76%.

n CoE was not downgraded for imprecision: Z-curve crossed the benefit boundary and sample size reached DARIS (382 vs. 184).

o CoE downgraded by two levels for Inconsistency: I ^2^ = 93%.

p CoE was not downgraded for imprecision: Z-curve crossed the benefit boundary and sample size reached DARIS (492 vs. 403).

#### Stricture Resolution and Technical Success

Seven RCTs involving 499 patients reported on stricture resolution. 19 27 29 30 31 32 33 Treatment with CSEMS likely results in a trivial increase to no difference in stricture resolution compared with treatment with MPS (RR: 1.04, 95% CI: 0.97–1.11; I ^2^ = 9%; moderate CoE). Results remained consistent with fixed-effects analysis, TSA, per-protocol analysis, and with subgroup analysis across different stricture etiologies. Sensitivity analysis considering a best-case scenario, where all missing CSEMS patients and none of the missing MPS patients were considered to develop an event, showed that treatment with CSEMS results in a statistically significant increase in the rate of stricture resolution compared with MPS (RR: 1.14, 95% CI: 1.03–1.25; I ^2^ = 37%; Table 2 , Supplementary Tables S3 and S4 , and Supplementary Figs. S3–S9 (available in the online version only)). 8

Two RCTs involving 50 patients reported on stricture resolution after initial treatment. 28 30 With high imprecision, treatment with CSEMS results in a trivial decrease to no difference in stricture resolution after initial treatment compared with treatment with MPS (RR: 0.84, 95% CI: 0.33–2.15; I ^2^ = 75%; very low CoE; Table 2 , Supplementary Tables S3 and S4 , and Supplementary Fig. S10 (available in the online version only)). 8

Three RCTs involving 307 patients reported on technical success. 19 27 32 Treatment with CSEMS likely results in no difference in technical success compared with treatment with MPS (RR: 1.00, 95% CI: 0.98–1.03; I ^2^ = 0%; moderate CoE). However, results are deemed imprecise according to TSA, and more trials are needed to reach a conclusion (RR: 1.00, TSA-adjusted 95% CI: 0.92–1.09; DARIS = 2,928; Table 2 , Supplementary Tables S3 and S4 , and Supplementary Figs. S11 and S12 (available in the online version only)). 8

#### Stricture Recurrence

Five RCTs involving 251 patients reported on stricture recurrence. 19 29 30 31 33 With high imprecision, CSEMS results in a clinically important increase in stricture recurrence compared with treatment with MPS (RR: 1.27, 95% CI: 0.66–2.45; I ^2^ = 31%; very low CoE). Results remained consistent with fixed-effects analysis, TSA, and with subgroup analysis across different stricture etiologies. Fixed-effects subgroup analysis in 4 RCTs involving 182 OLT patients showed a statistically significant increase in the rate of stricture recurrence in the group receiving CSEMS compared with those receiving MPS (RR: 2.15, 95% CI: 1.08–4.27; I ^2^ = 48%). However, the results are highly imprecise ( Table 2 , Supplementary Tables S3 and S4 , and Supplementary Figs. S13 and S14 (available in the online version only)). 8 19 30 31 33

#### Adverse Events, All-Cause Mortality, and Stent Migration

Seven RCTs involving 499 patients reported on the incidence of adverse events. 19 27 29 30 31 32 33 With high imprecision, treatment with CSEMS results in a trivial increase or no difference in adverse events compared with treatment with MPS (IRR: 1.13, 95% CI: 0.86–1.47; I ^2^ = 5%; very low CoE). Results remained consistent with fixed-effects analysis, TSA, and with subgroup analysis across different stricture etiologies ( Table 2 , Supplementary Tables S3 and S4 , and Supplementary Figs. S15 and S16 (available in the online version only)). Supplementary Table S5 (available in the online version only) lists all reported adverse events in the included RCTs. 8

Seven RCTs involving 501 patients reported on all-cause mortality. 19 27 29 30 31 32 33 With high imprecision, treatment with CSEMS results in a trivial increase or no difference in all-cause mortality compared with treatment with MPS (RR: 1.22, 95% CI: 0.41–3.62; I ^2^ = 25%; very low CoE). Results remained consistent with fixed-effects analysis, TSA, and with subgroup analysis across different stricture etiologies ( Table 2 , Supplementary Tables S3 and S4 , and Supplementary Fig. S17 (available in the online version only)). 8

Seven RCTs involving 499 patients reported on the incidence of stent migration. 19 27 29 30 31 32 33 With high imprecision, treatment with CSEMS results in a trivial increase or no difference in stent migration compared with treatment with MPS (IRR: 1.13, 95% CI: 0.72–1.78; I ^2^ = 12%; very low CoE). Results remained consistent with fixed-effects analysis, TSA, and with subgroup analysis across different stricture etiologies ( Table 2 , Supplementary Tables S3 and S4 , and Supplementary Figs. S18 and S19 (available in the online version only)). 8

#### Number of ERCP Procedures, Number of Stents Deployed, and Total Stent Treatment Duration

Five RCTs involving 382 patients reported on the number of ERCP procedures. 19 30 31 32 33 A total of 314 ERCP procedures were performed in the CSMES group versus 497 procedures in the MPS group. Treatment with CSEMS might require fewer ERCP procedures compared with treatment with MPS (MD: −1.84, 95% CI: −2.56 to −1.11; I ^2^ = 76%; low CoE). Results remained consistent with fixed effects analysis, TSA, and with subgroup analysis across different stricture etiologies ( Table 2 , Supplementary Tables S3 and S4 , and Supplementary Figs. S20–S22 ) (available in the online version only). 8

Seven RCTs involving 492 patients reported on the number of stents deployed. 19 27 29 30 31 32 33 A total of 348 stents were deployed in the CSMES group versus 1,661 stents in the MPS group. However, we are uncertain whether treatment with CSEMS requires fewer stents compared with treatment with MPS (MD: −6.04, 95% CI: −9.55 to −2.52; I ^2^ = 93%; very low CoE). Results remained consistent with fixed-effects analysis, TSA, and with subgroup analysis across different stricture etiologies ( Table 2 , Supplementary Tables S3 and S4 , and Supplementary Figs. S23 and S24 ) (available in the online version only). 8

Five RCTs involving 382 patients reported on the total duration of stent treatment. 19 30 31 32 33 Treatment with CSEMS requires a shorter duration of stent treatment compared with treatment with MPS, 242 days versus 305 days, respectively, (MD: −119.02 days, 95% CI: −137.41 to −100.62; I ^2^ = 96%; very low CoE). Results remained consistent with fixed-effects analysis, TSA, and with subgroup analysis in OLT patients. In patients with CP, treatment with CSEMS required a statistically nonsignificant shorter duration of stent treatment compared with treatment with MPS (MD: −18.68 days, 95% CI: −62.72 to 25.35; I ^2^ = 44%; Table 2 , Supplementary Tables S3 and S4 , and Supplementary Figs. S25–S27 (available in the online version only)). 8

#### Health Care Costs

Three RCTs involving 109 patients reported on treatment costs. 28 30 31 Savings in treatment costs ranged between 41 and 57% in favor of CSEMS over MPS. Kaffes et al reported 56% savings in costs of completing the treatment in patients receiving CSEMS compared with those receiving MPS ($12,913 vs. $29,280 Australian dollars; 20 patients; *p* = 0.08). 30 Martins et al reported 57% savings in costs in patients receiving CSEMS compared with those receiving MPS ($6,903 vs. $16,095; 59 patients; *p* < 0.01). 31 Cantù et al reported no statistically significant difference in treatment costs between patients receiving CSEMS or MPS (€10,490 ± 6,709 vs. €9,550 ± 2,497; 30 patients; *p* = 0.4). However, CSEMSs were more cost-effective than MPSs when considering patients who achieved and maintained clinical remission after first-line treatment with 41% savings in costs (€5,371 ± 237 vs. €9,092 ± 1,544; 20 patients; *p* < 0.001). 28 Majority of treatment costs were due to endoscopy (>70%), followed by hospitalization-related costs (>20%). CoE was very low due to study limitations and severe imprecision.

## Discussion

### Summary

With CoE ranging from very low to moderate, our study suggests that CSEMS and MPS have comparable efficacy and safety profiles. However, CSEMS required significantly a smaller number of ERCP procedures, stents, and shorter therapy duration, averaging 2 fewer procedures, 6 fewer stents, and 120 fewer days. Three RCTs showed that CSEMSs are more cost-effective than MPS, with savings in health care costs ranging from 41 to 57%. 28 30 31 A cost-effectiveness analysis study used a decision-analytic Markov model to simulate a cohort of 50-year-old patients with BBS due to CP over a 5-year period. 34 The study compared MPS with 3-month exchanges to CSEMS with 6-month or 12-month indwelling periods. The model's input parameters were based on incidence data compiled through a systematic literature review, which aligned with the rates in our systematic review. The study concluded that CSEMSs with 6-month or 12-month indwelling periods are more cost-effective than MPSs, with CSEMS with a 12-month indwelling period being the most cost-effective strategy. Compared with MPS, CSEMS with a 12-month indwelling period achieved average savings of $17,649 or 61.7%.

Our findings align with previous meta-analyses. 5 35 36 37 However, Giri et al and Kamal et al included eight RCTs in their analyses, treating Tal et al and Cantù et al as separate trials, which resulted in double-counting the same population and potentially introducing bias. 28 33 36 37 This is evident in Kamal et al, which reported a significantly higher stent migration rate in the CSEMS group (RR: 2.41, 95% CI: 1.11–5.25). 37 This discrepancy may also stem from inconsistencies in the unit of analysis, as some studies pooled outcome data per patient and others per stent or ERCP procedure.

Our evidence synthesis agrees with recommendations from several guidelines, including the ACG, 4 Asia–Pacific consensus guidelines, 38 the European Society of Gastrointestinal Endoscopy (ESGE), 39 and the international consensus statements for endoscopic management of distal biliary stricture. 40 All guidelines emphasize the comparability of the two stent modalities. However, the ACG guideline favors CSEMS over MPS based on feasibility, suggesting that a 12-month indwelling period for CSEMS is more favorable than a 6-month period. 4 The ESGE guideline recommends a 6-month indwelling period for CSEMS, which may be less cost-effective than a 12-month period. 39 Future updates of the ESGE guidelines should reflect this change, as CSEMSs with a 12-month indwelling period are likely to be more cost-effective. Most recommendations are derived from low-quality evidence and are therefore conditional.

MPSs still play a crucial role in managing BBSs in specific scenarios: when the stricture is less than 2 cm from the hepatic hilum, when the gallbladder is in situ and the cystic duct orifice cannot be avoided by CSEMS, when CSEMS has migrated, or not been tolerated by the patient, or when there is recurrence of BBS after CSEMS removal. 4

Our study has several notable strengths. We adhered to the PRISMA 2020 guidelines throughout the process of this systematic review. To reduce bias in our findings, we exclusively included RCTs. Our comprehensive literature search utilized multiple databases and controlled vocabulary, ensuring a highly sensitive search process. We conducted meta-analyses using a conservative restricted maximum likelihood random-effects model, along with sensitivity analyses, to assess the robustness of our results. Additionally, we examined the impact of different BBS etiologies through subgroup analyses. To further minimize the risk of false positives due to sparse data and repeated analyses, we applied TSA. 14 41 Our review also performed sample size calculations to guide the design of adequately powered future research. In contrast to prior studies, we set criteria to evaluate the CoE using the GRADE framework. Finally, we investigated the health care costs associated with each type of stent, an important consideration when choosing between therapies with comparable efficacy and safety profiles.

## Limitations

Some limitations are noted in our systematic review. There is heterogeneity in our results stemming from the variable etiologies and severity of BBS, different types and sizes of stents used, duration of indwelling stent, interval at which stents are exchanged, and use of balloon dilatation. Subgroup meta-analysis based on etiology was not adequate to explain the observed heterogeneity. Many variables were not sufficiently reported by the primary studies, and we were not able to conduct meta-regression or publication bias assessment due to the small number of included RCTs. TSA suggested that many of the analyses were underpowered to detect a minimally important difference between the two modes of therapy, including stricture resolution after initial intervention, stricture recurrence, stent migration, all-cause mortality, and adverse events. Health care costs were reported by three RCTs only, and a meta-analysis was not feasible due to the inherent heterogeneity between the studies, given the difference in health care systems, local regulations, insurance coverage, currencies, and associated costs, among other variables. 28 30 31

## Conclusion

Our analysis, with CoE ranging from very low to moderate, suggests that CSEMSs are likely comparable to MPSs in terms of efficacy and safety. However, the data indicate that CSEMSs may be more cost-effective than MPSs. To validate the safety profile, determine the feasibility of these two stents, and to inform strong recommendations, further trials and cost-effectiveness analyses are necessary. The sample size calculations from our analyses can guide the design of these future trials.

## References

[1] Wanjara S Kashyap S Bile Duct StricturePubMed. Published 2021.https://www.ncbi.nlm.nih.gov/books/NBK559217/

[2] Kapoor B S Mauri G Lorenz J M Management of biliary strictures: state-of-the-art review Radiology 2018 289 03 590 603 30351249 10.1148/radiol.2018172424

[3] Bowlus C L Olson K A Gershwin M E Evaluation of indeterminate biliary strictures Nat Rev Gastroenterol Hepatol 2016 13 01 28 37 26526122 10.1038/nrgastro.2015.182

[4] Elmunzer B J Maranki J L Gómez V ACG clinical guideline: diagnosis and management of biliary strictures Am J Gastroenterol 2023 118 03 405 426 36863037 10.14309/ajg.0000000000002190

[5] Khan M A Baron T H Kamal F Efficacy of self-expandable metal stents in management of benign biliary strictures and comparison with multiple plastic stents: a meta-analysis Endoscopy 2017 49 07 682 694 28561199 10.1055/s-0043-109865

[6] Benign Biliary Stenoses Working Group Lakhtakia S Reddy N Dolak W Long-term outcomes after temporary placement of a self-expanding fully covered metal stent for benign biliary strictures secondary to chronic pancreatitis Gastrointest Endosc 2020 91 02 361 369000 31494135 10.1016/j.gie.2019.08.037

[7] Page M J McKenzie J E Bossuyt P M The PRISMA 2020 statement: an updated guideline for reporting systematic reviews BMJ 2021 372 n71 33782057 10.1136/bmj.n71 PMC8005924

[8] Nayfeh T Supplementary materialMay 2025. 10.6084/m9.figshare.29182847.v2

[9] Sterne J AC Savović J Page M J RoB 2: a revised tool for assessing risk of bias in randomised trials BMJ 2019 366 l4898 31462531 10.1136/bmj.l4898

[10] Hozo S P Djulbegovic B Hozo I Estimating the mean and variance from the median, range, and the size of a sample BMC Med Res Methodol 2005 5 13 15840177 10.1186/1471-2288-5-13 PMC1097734

[11] Deeks J J Higgins J P Altman D G Analysing data and undertaking meta-analyses Cochrane Handbook for Systematic Reviews of InterventionsPublished online September 20,2019 241 284

[12] Page M J Higgins J P Sterne J A Assessing risk of bias due to missing results in a synthesis Cochrane Handbook for Systematic Reviews of InterventionsPublished online September 20,2019 349 374

[13] Wetterslev J Jakobsen J C Gluud C Trial Sequential Analysis in systematic reviews with meta-analysis BMC Med Res Methodol 2017 17 01 39 28264661 10.1186/s12874-017-0315-7 PMC5397700

[14] Wetterslev J Thorlund K Brok J Gluud C Trial sequential analysis may establish when firm evidence is reached in cumulative meta-analysis J Clin Epidemiol 2008 61 01 64 75 18083463 10.1016/j.jclinepi.2007.03.013

[15] Wetterslev J Thorlund K Brok J Gluud C Estimating required information size by quantifying diversity in random-effects model meta-analyses BMC Med Res Methodol 2009 9 86 20042080 10.1186/1471-2288-9-86 PMC2809074

[16] Jakobsen J C Wetterslev J Winkel P Lange T Gluud C Thresholds for statistical and clinical significance in systematic reviews with meta-analytic methods BMC Med Res Methodol 2014 14 120 25416419 10.1186/1471-2288-14-120 PMC4251848

[17] Castellini G Bruschettini M Gianola S Gluud C Moja L Assessing imprecision in Cochrane systematic reviews: a comparison of GRADE and Trial Sequential Analysis Syst Rev 2018 7 01 110 30055658 10.1186/s13643-018-0770-1 PMC6064621

[18] Thorlund K EJ Wetterslev J Brok J Imberger G Gluud C User Manual for Trial Sequential Analysis (TSA)2nd ed.2017 1 119. Accessed January 30, 2025 at:https://ctu.dk/tsa

[19] Coté G A Slivka A Tarnasky P Effect of covered metallic stents compared with plastic stents on benign biliary stricture resolution: a randomized clinical trial JAMA 2016 315 12 1250 1257 27002446 10.1001/jama.2016.2619 PMC5544902

[20] Balshem H Helfand M Schünemann H J GRADE guidelines: 3. Rating the quality of evidence J Clin Epidemiol 2011 64 04 401 406 21208779 10.1016/j.jclinepi.2010.07.015

[21] Guyatt G H Oxman A D Kunz R GRADE guidelines 6. Rating the quality of evidence–imprecision J Clin Epidemiol 2011 64 12 1283 1293 21839614 10.1016/j.jclinepi.2011.01.012

[22] GRADE Working Group Guyatt G H Oxman A D Kunz R GRADE guidelines: 8. Rating the quality of evidence–indirectness J Clin Epidemiol 2011 64 12 1303 1310 21802903 10.1016/j.jclinepi.2011.04.014

[23] GRADE Working Group Guyatt G H Oxman A D Kunz R GRADE guidelines: 7. Rating the quality of evidence–inconsistency J Clin Epidemiol 2011 64 12 1294 1302 21803546 10.1016/j.jclinepi.2011.03.017

[24] Guyatt G H Oxman A D Montori V GRADE guidelines: 5. Rating the quality of evidence–publication bias J Clin Epidemiol 2011 64 12 1277 1282 21802904 10.1016/j.jclinepi.2011.01.011

[25] Guyatt G H Oxman A D Vist G GRADE guidelines: 4. Rating the quality of evidence–study limitations (risk of bias) J Clin Epidemiol 2011 64 04 407 415 21247734 10.1016/j.jclinepi.2010.07.017

[26] Zeng L Brignardello-Petersen R Hultcrantz M GRADE Guidance 34: update on rating imprecision using a minimally contextualized approach J Clin Epidemiol 2022 150 216 224 35934265 10.1016/j.jclinepi.2022.07.014

[27] Artifon E L Coelho F Frazao M A prospective randomized study comparing partially covered metal stent versus plastic multistent in the endoscopic management of patients with postoperative benign bile duct strictures: a follow-up above 5 years Rev Gastroenterol Peru 2012 32 01 26 31 22476175

[28] Cantù P Santi G Rosa R Cost analysis of a long-term randomized controlled study in biliary duct-to-duct anastomotic stricture after liver transplantation Transpl Int 2021 34 05 825 834 33730421 10.1111/tri.13867

[29] Haapamäki C Kylänpää L Udd M Randomized multicenter study of multiple plastic stents vs. covered self-expandable metallic stent in the treatment of biliary stricture in chronic pancreatitis Endoscopy 2015 47 07 605 610 25590182 10.1055/s-0034-1391331

[30] Kaffes A Griffin S Vaughan R A randomized trial of a fully covered self-expandable metallic stent versus plastic stents in anastomotic biliary strictures after liver transplantation Therap Adv Gastroenterol 2014 7 02 64 71 24587819 10.1177/1756283X13503614 PMC3903084

[31] Martins F P De Paulo G A Contini M LC Ferrari A P Metal versus plastic stents for anastomotic biliary strictures after liver transplantation: a randomized controlled trial Gastrointest Endosc 2018 87 01 1310 1.31E15 28455159 10.1016/j.gie.2017.04.013

[32] Ramchandani M Lakhtakia S Costamagna G Fully covered self-expanding metal stent vs multiple plastic stents to treat benign biliary strictures secondary to chronic pancreatitis: a multicenter randomized trial Gastroenterology 2021 161 01 185 195 33741314 10.1053/j.gastro.2021.03.015

[33] Tal A O Finkelmeier F Filmann N Multiple plastic stents versus covered metal stent for treatment of anastomotic biliary strictures after liver transplantation: a prospective, randomized, multicenter trial Gastrointest Endosc 2017 86 06 1038 1045 28302527 10.1016/j.gie.2017.03.009

[34] Thiruvengadam N R Saumoy M Schneider Y Kochman M L Fully covered self-expanding stents are cost-effective at remediating biliary strictures in patients with chronic pancreatitis Clin Gastroenterol Hepatol 2023 21 02 552 5.54E6 35181569 10.1016/j.cgh.2022.02.019

[35] Zhang X Wang X Wang L Tang R Dong J Effect of covered self-expanding metal stents compared with multiple plastic stents on benign biliary stricture: a meta-analysis Medicine (Baltimore) 2018 97 36 e12039 30200083 10.1097/MD.0000000000012039 PMC6133465

[36] Giri S Jearth V Sundaram S Covered self-expanding metal stents versus multiple plastic stents for benign biliary strictures: an updated meta-analysis of randomized controlled trials Cureus 2022 14 04 e24588 35651420 10.7759/cureus.24588 PMC9138190

[37] Kamal F Ali Khan M Lee-Smith W Metal versus plastic stents in the management of benign biliary strictures: systematic review and meta-analysis of randomized controlled trials Eur J Gastroenterol Hepatol 2022 34 05 478 487 35170533 10.1097/MEG.0000000000002352

[38] Hu B Sun B Cai Q Asia-Pacific consensus guidelines for endoscopic management of benign biliary strictures Gastrointest Endosc 2017 86 01 44 58 28283322 10.1016/j.gie.2017.02.031

[39] Dumonceau J M Tringali A Papanikolaou I S Endoscopic biliary stenting: indications, choice of stents, and results: European Society of Gastrointestinal Endoscopy (ESGE) Clinical Guideline - Updated October 2017 Endoscopy 2018 50 09 910 930 30086596 10.1055/a-0659-9864

[40] Nakai Y Isayama H Wang H P International consensus statements for endoscopic management of distal biliary stricture J Gastroenterol Hepatol 2020 35 06 967 979 31802537 10.1111/jgh.14955 PMC7318125

[41] Brok J Thorlund K Gluud C Wetterslev J Trial sequential analysis reveals insufficient information size and potentially false positive results in many meta-analyses J Clin Epidemiol 2008 61 08 763 769 18411040 10.1016/j.jclinepi.2007.10.007

