# Supplementary material for: Covered Self-Expandable Metallic Stents versus Multiple Plastic Stents for Benign Biliary Strictures: A Systematic Review, Meta-Analysis, and Trial Sequential Analysis
Source: Avicenna J Med. 2026 Mar 24;16(1):16–26. doi: 10.1055/s-0046-1818623 (PMC13288589; doi:10.1055/s-0046-1818623)

Supplementary Materials

Table of Contents

Table 1 Etiologies of Benign Biliary Strictures 3

Search Strategies 4

Table 2 Study baseline characteristics 9

Fig. 1 Risk of bias assessment based on (RoB2 tool) barplot showing overall quality of studies across different domains 15

Fig. 2 Risk of bias assessment based on (RoB2 tool) traffic light showing quality of individual studies across different domains 16

Table 3 Outcomes 17

Table 4 Trial Sequential Analysis 21

Table 5 Adverse events 22

Fig. 3 Pooled estimate of stricture resolution – intention to treat analysis 25

Fig. 4 Pooled estimate of stricture resolution – per protocol analysis 26

Fig. 5 Pooled estimate of stricture resolution – best case scenario 27

Fig. 6 Pooled estimate of stricture resolution – worst case scenario 28

Fig. 7 Pooled estimate of stricture resolution – subgroup meta-analysis based on etiology- intention to treat analysis 29

Fig. 8 Pooled estimate of stricture resolution – subgroup meta-analysis based on etiology – per protocol analysis 30

Fig. 9 Trial sequential analysis of stricture resolution 31

Fig. 10 Pooled estimate of stricture resolution after initial treatment 32

Fig. 11 Pooled estimate of technical success 33

Fig. 12 Trial sequential analysis of technical success 34

Fig. 13 Pooled estimate of stricture recurrence 35

Fig. 14 Pooled estimate of stricture recurrence – subgroup analysis based on etiology 36

Fig. 15 Pooled estimate of adverse events - subgroup meta-analysis based on etiology 37

Fig. 16 Trial sequential analysis of adverse events 38

Fig. 17 Pooled estimate of all-cause mortality 39

Fig. 18 Pooled estimate of stent migration 40

Fig. 19 Trial sequential analysis of stent migration 41

Fig. 20 Pooled estimate of number of ERCP 42
Fig. 21 Pooled estimate of number of ERCP - subgroup analysis based on etiology 43
Fig. 22 Trial sequential analysis of number of ERCP 44
Fig. 23 Pooled estimate of number of stents – subgroup analysis based on etiology 45
Fig. 24 Trial sequential analysis of number of stents 46
Fig. 25 Pooled estimate of number of stent treatment duration 47
Fig. 26 Pooled estimate of stent treatment duration – subgroup analysis based on etiology 48
Fig. 27 Trial sequential analysis of stent treatment duration 49

Table 1 Etiologies of Benign Biliary Strictures

Benign		Malignant
Iatrogenic	• Cholecystectomy<br>• Postendoscopic sphincterotomy<br>• Posthepatobiliary surgery (e.g. Whipple procedure, and OLT including anastomotic and non-anastomotic strictures)<br>• Local cancer treatment (chemoembolization, radiation therapy, microwave ablation, and radiofrequency ablation)	Pancreatic adenocarcinoma
Autoimmune diseases	• PSC<br>• Autoimmune (IgG4–mediated) pancreatitis<br>• IgG4-associated cholangitis<br>• Sarcoidosis<br>• Eosinophilic cholangitis<br>• Mast cell cholangitis<br>• Histiocytosis X	Cholangiocarcinoma (Sporadic or PSC-associated)
Infections	• Tuberculosis<br>• Oriental cholangiohepatitis (Ascaris lumbricoides and Clonorchis sinensis)<br>• Parasitic<br>• Acute cholangitis<br>• Recurrent pyogenic cholangitis<br>• HIV-cholangiopathy	Ampullary adenocarcinoma and periampullary cancer (e.g. duodenal cancer)
Acute or chronic pancreatitis		Gallbladder carcinoma
Cholelithiasis (Mirizzi syndrome)		Hepatocellular carcinoma
Vascular	• Ischemic cholangiopathy<br>• Vasculitis<br>• Intra-arterial chemotherapy<br>• Portal hypertensive biliopathy<br>• Hepatic artery stenosis or thrombosis	Lymphoma
Miscellaneous	• Blunt and penetrating abdominal trauma<br>• Biliary inflammatory pseudotumors<br>• Extrinsic compression by a pancreatic fluid collection<br>• Papillary stenosis	Metastasis to regional solid organs and lymph nodes (e.g. intra- and extra- hepatic metastasis including colon, breast, renal cell cancer, and rarely squamous cell carcinoma)

OLT: Orthotopic liver transplantation; PSC: Primary sclerosing cholangitis

Search Strategies

OVID

Database(s): Ovid MEDLINE(R) 1946 to Present and Epub Ahead of Print, In-Process & Other Non-Indexed Citations and Ovid MEDLINE(R) Daily, EBM Reviews - Cochrane Central Register of Controlled Trials May 2024, EBM Reviews - Cochrane Database of Systematic Reviews 2005 to June 5, 2024, Embase 1974 to 2024 June 05
Search Strategy:

#	Searches
1	(benign* or "non-cancerous" or noncancerous or "non-malignant" or nonmalignant or post-op* or postop* or post-surg* or postsurg* or anastomo* or liver-transplant* or chronic-pancreat* or cholecystectom* or pancreatitis or cholangitis or cholangiohepatitis or "mirizzi-syndrome" or radiation or (blunt adj5 abdomin* adj5 trauma*) or "portal-biliopath*" or "polyarteritis-nodosa" or "systemic-lupus-erythematosus" or tuberculosis or histoplasmosis or "sphincter-of-Oddi" or "papillary-stenosis" or "choledochal-cyst*").ti,ab,hw,kf.
2	"Pancreatitis, Chronic"/
3	"Postoperative Complications"/
4	"Anastomosis, Surgical"/
5	"Liver Transplantation"/
6	Choledochal Cyst/
7	Cholangitis, Sclerosing/
8	or/1-7
9	exp Bile Ducts/
10	("bile-duct*" or "bile-tract" or biliary).ti,ab,hw,kf.
11	or/9-10
12	Constriction, Pathologic/
13	(stricture* or obstruct* or constrict* or occlusion* or occlud* or stenos* or stenotic).ti,ab,kf.
14	or/12-13
15	11 and 14
16	exp Cholestasis/ or cholestasis.ti,ab,kf.
17	15 or 16
18	8 and 17
19	benign biliary stricture/ or (benign adj5 (bile* or biliary) adj5 (stricture* or obstruct* or constrict* or occlus* or occlud* or stenos* or stenotic)).ti,ab.

20	18 or 19
21	Self Expandable Metallic Stents/ or exp *metal stent/
22	((metal* or alloy* or steel or nitinol or niti or nickel or titanium) adj5 stent*).ti,ab. or (wallflex or wallstent or hanarostent or hanaro or bonastent or SEMS* or "C-SEMS*" or CSEMS* or FCSEMS* or "FC-SEMS*" or USEMS*).ti,ab,kf.
23	(exp *Metals/ or (metal* or alloy* or steel or nitinol or niti or nickel or titanium).ti,ab,hw,kf.) and (*Stents/ or *stent/ or stent*.ti.)
24	21 or 22 or 23
25	*plastic stent/ or ((exp *Plastics/ or (plastic* or microplastic* or nylon* or polypropylene* or polystyrene* or (resin* adj5 synthetic*) or polymer* or Polytetrafluoroethylene* or polyurethan* or polyester* or polyethylene* or polyvinyl* or biopolymer*).ti,ab,kf.) and (*Stents/ or *stent/ or stent*.ti.))
26	((plastic* or microplastic* or nylon* or polypropylene* or polystyrene* or (resin* adj5 synthetic*) or polymer* or Polytetrafluoroethylene* or polyurethan* or polyester* or polyethylene* or polyvinyl* or biopolymer*) adj5 stent*).ti,ab.
27	25 or 26
28	exp Randomized Controlled Trial/
29	exp Double-Blind Method/ or double blind procedure/
30	exp Single-Blind Method/
31	exp Placebos/
32	exp Placebo Effect/
33	randomization/
34	((randomized adj3 study) or (randomized adj3 trial) or (randomised adj3 study) or (randomised adj3 trial) or "pragmatic clinical trial" or (doubl* adj1 blind*) or (doubl* adj1 mask*) or (singl* adj blind*) or (singl* adj mask*) or (tripl* adj blind*) or (tripl* adj mask*) or (trebl* adj blind*) or (trebl* adj mask*) or "latin square" or placebo* or nocebo* or random*).mp,pt.
35	exp clinical trials/ or controlled clinical trial.pt. or controlled clinical trial/ or "randomized controlled trial"/ or exp clinical trial/ or exp major clinical study/ or exp prospective study/ or exp retrospective study/ or comparative study/ or exp cohort studies/ or exp case control study/ or exp case study/ or exp intervention study/ or intermethod comparison/
36	(("phase-0" or "phase-1" or "phase-I" or "phase-2" or "phase-II" or "phase-3" or "phase-III" or "phase-4" or "phase-IV" or "phase-i" or "phase-ii" or "phase-iii" or "phase-iv") adj3 (trial or study or studies)).mp,pt.
37	((clinical or observational or cohort or multicenter or multicentre or retrospective or prospective* or "cross-sectional" or case or random* or pilot or controlled) adj3 (trial* or study or studies or series or design or report or reports)).ti,ab. or clinical trial.ot. or ((enroll* or clinical) and (study or trial)).mp. or (open adj label).ti,ab. or ("cross-over" or crossover).ti,ab.
38	or/28-36

39	20 and 24 and 27 and 38
40	(exp animals/ or exp nonhuman/) not exp humans/
41	((alpaca or alpacas or algae* or amphibian or amphibians or animal or animals or antelope or armadillo or armadillos or avian or baboon or baboons or bats or beagle or beagles or bee or bees or bird or birds or bison or bovine or buffalo or buffaloes or buffalos or "c elegans" or "Caenorhabditis elegans" or camel or camels or canine or canines or canis or carp or cats or catfish or cattle or chamaeleo* or chameleon* or chick or chicken or chickens or chicks or chimp or chimpanze or chimpanzees or chimps or cow or cows or "D melanogaster" or "dairy calf" or "dairy calves" or deer or dog or dogs or donkey or donkeys or drosophila or "Drosophila melanogaster" or duck or duckling or ducklings or ducks or equid or equids or equine or equines or feline or felines or ferret or ferrets or finch or finches or fish or flatworm or flatworms or fox or foxes or frog or frogs or "fruit flies" or "fruit fly" or "G mellonella" or "Galleria mellonella" or geese or gerbil or gerbils or goat or goats or goose or gorilla or gorillas or groundhog or groundhogs or hamster or hamsters or hare or hares or heifer or heifers or horse or horses or iguana or iguanas or insect or insects or jellyfish or kangaroo or kangaroos or kitten or kittens or "laboratory animal*" or lagomorph or lagomorphs or lamb or lambs or lemur or lemurs or lemuridae or llama or llamas or macaque or macaques or macaw or macaws or marmoset or marmosets or mice or minipig or minipigs or mink or minks or monkey or monkeys or mouse or mule or mules or muskrat or muskrats or nematode or nematodes or newt or newts or octopus or octopuses or orangutan or "orang-utan" or orangutans or "orang-utans" or oxen or parrot or parrots or pig or pigeon or pigeons or piglet or piglets or pigs or porcine or primate or primates or poultry or quail or rabbit or rabbits or rat or rats or reptile or reptiles or rodent or rodents or ruminant or ruminants or salmon or sheep or shrimp or slug or slugs or swine or tamarin or tamarins or tilapia or tilapias or toad or toads or trout or urchin or urchins or vole or voles or waxworm or waxworms or weasel or weasels or wolf or wolves or worm or worms or wrass* or xenopus or "zebra fish" or zebrafish) not (human or humans or patient or patients)).ti,ab,hw,kf.
42	(rat or rats or mice or mouse or murine or pig or pigs or porcine or swine or dog or dogs).ti.
43	or/40-42
44	39 not 43
45	(conference abstract or conference review or editorial or erratum or note or addresses or autobiography or bibliography or biography or blogs or comment or dictionary or directory or interactive tutorial or lectures or legal cases or legislation or news or newspaper article or patient education handout or periodical index or portraits or published erratum or video-audio media or webcasts).mp. or conference abstract.st.
46	44 not 45
47	46 not ("case-reports" or review or comment or editorial or guideline or news).pt.
48	limit 47 to english language [Limit not valid in CDSR; records were retained]
49	remove duplicates from 48

SCOPUS

1 (TITLE-ABS-KEY ((plastic* OR microplastic* OR nylon* OR polypropylene* OR polystyrene* OR (resin* W/5 synthetic*) OR polymer* OR polytetrafluoroethylene* OR polyurethan* OR polyester* OR polyethylene* OR polyvinyl* OR biopolymer*) W/5 stent*)) AND (TITLE-ABS-KEY ((metal* OR alloy* OR steel OR nitinol OR niti OR nickel OR titanium) W/5 stent*) OR TITLE-ABS-KEY (wallflex OR wallstent OR hanarostent OR hanaro OR bonastent OR sems* OR "C-SEMS*" OR csems* OR fcsems* OR "FC-SEMS*" OR usems*)) AND (TITLE-ABS-KEY ("bile-duct*" OR "bile-tract" OR biliary) W/7 (stricture* OR obstruct* OR constrict* OR occlusion* OR occlud* OR stenos* OR stenotic) OR TITLE-ABS-KEY (cholestasis)) AND (TITLE-ABS-KEY (benign* OR "non-cancerous" OR noncancerous OR "non-malignant" OR nonmalignant OR post-op* OR postop* OR post-surg* OR postsurg* OR anastomo* OR liver-transplant* OR chronic-pancreat* OR cholecystectom* OR pancreatitis OR cholangitis OR cholangiohepatitis OR "mirizzi-syndrome" OR radiation OR (blunt W/5 abdomin* W/5 trauma*) OR "portal-biliopath*" OR "polyarteritis-nodosa" OR "systemic-lupus-erythematosus" OR tuberculosis OR histoplasmosis OR "sphincter-of-Oddi" OR "papillary-stenosis" OR "choledochal-cyst*"))

2 TITLE-ABS-KEY ((control* W/3 study) OR (control* W/3 trial) OR (randomized W/3 study) OR (randomized W/3 trial) OR (randomized W/3 study) OR (randomized W/3 trial) OR "pragmatic-clinical-trial" OR (doubl* W/1 blind*) OR (doubl* W/1 mask*) OR (singl* W/1 blind*) OR (singl* W/1 mask*) OR (tripl* W/1 blind*) OR (tripl* W/1 mask*) OR (trebl* W/1 blind*) OR (trebl* W/1 mask*) OR "latinsquare" OR placebo* OR nocebo*or AND random*)

3 1 and 2

4 INDEX(embase) OR INDEX(medline) OR PMID(0* OR 1* OR 2* OR 3* OR 4* OR 5* OR 6* OR 7* OR 8* OR 9*)

5 3 not 4

6 (TITLE-ABS-KEY ((alpaca OR alpacas OR amphibian OR amphibians OR animal OR animals OR antelope OR armadillo OR armadillos OR avian OR baboon OR baboons OR beagle OR beagles OR bee OR bees OR bird OR birds OR bison OR bovine OR

buffalo OR buffaloes OR buffalos OR "c elegans" OR "Caenorhabditis elegans" OR camel OR camels OR canine OR canines OR carp OR cats OR cattle OR chick OR chicken OR chickens OR chicks OR chimp OR chimpanze OR chimpanzees OR chimps OR cow OR cows OR "D melanogaster" OR "dairy calf" OR "dairy calves" OR deer OR dog OR dogs OR donkey OR donkeys OR drosophila OR "Drosophila melanogaster" OR duck OR duckling OR ducklings OR ducks OR equid OR equids OR equine OR equines OR feline OR felines OR ferret OR ferrets OR finch OR finches OR fish OR flatworm OR flatworms OR fox OR foxes OR frog OR frogs OR "fruit flies" OR "fruit fly" OR "G mellonella" OR "Galleria mellonella" OR geese OR gerbil OR gerbils OR goat OR goats OR goose OR gorilla OR gorillas OR hamster OR hamsters OR hare OR hares OR heifer OR heifers OR horse OR horses OR insect OR insects OR jellyfish OR kangaroo OR kangaroos OR kitten OR kittens OR lagomorph OR lagomorphs OR lamb OR lambs OR llama OR llamas OR macaque OR macaques OR macaw OR macaws OR marmoset OR marmosets OR mice OR minipig OR minipigs OR mink OR minks OR monkey OR monkeys OR mouse OR mule OR mules OR nematode OR nematodes OR octopus OR octopuses OR orangutan OR "orang-utan" OR orangutans OR "orang-utans" OR oxen OR parrot OR parrots OR pig OR pigeon OR pigeons OR piglet OR piglets OR pigs OR porcine OR primate OR primates OR quail OR rabbit OR rabbits OR rat OR rats OR reptile OR reptiles OR rodent OR rodents OR ruminant OR ruminants OR salmon OR sheep OR shrimp OR slug OR slugs OR swine OR tamarin OR tamarins OR toad OR toads OR trout OR urchin OR urchins OR vole OR voles OR waxworm OR waxworms OR worm OR worms OR xenopus OR "zebra fish" OR zebrafish) AND NOT (human OR humans OR patient OR patients)))

7 5 not 6

8 LIMIT-TO (SRCTYPE , "j") AND LIMIT-TO (LANGUAGE , "English") AND LIMIT-TO (DOCTYPE , "ar")

Table 2 Study baseline characteristics

Author, Year	Inclusion criteria	Exclusion criteria	Details of CSEMS	Details of MPS	Funding sources
Artifon, 2012	• Patients aged ≥ 18<br>• Diagnosed with BBS based on MRI, intraoperative cholangiography, and ERCP	NR	• A guidewire was passed through the stenosis followed by the dilation of a 4-mm to 6-mm balloon (Boston Medical Scientific, Natick, USA),<br>• Biliary partially CSEMS (Boston Medical Scientific, Natick, USA, Bloomington, USA) were deployed	• A guidewire was passed through the stenosis followed by the dilation of a 4-mm to 6-mm balloon (Boston Medical Scientific, Natick, USA),<br>• 8.5-Fr and/or 10-Fr MPSs 7 cm and/or 9 cm long (Boston Medical Scientific, Natick, USA, Bloomington, USA) were deployed	NR
Cantù, 2021	• LT patients with AS suspected by ≥ 3 months of abnormal in liver tests<br>• Duct-to-duct anastomosis narrowing on MR or Kehr cholangiography consistent with AS	• LT patients with recurrent AS after any previous endoscopic treatment<br>• Patients with hepatico-jejunal anastomosis	• After guide-wired cannulation of the CBD, occlusive cholangiography was performed, and the AS confirmed<br>• A fully CSEMS was used<br>• No balloon dilation was needed to place fully CSEMSs<br>• The fully CSEMS was removed after 6 months<br>• The diameter of the fully CSEMS (8 or 10 mm) was chosen according to the diameters of the native and donor CBDs	• After guide-wired cannulation of the CBD, occlusive cholangiography was performed, and the AS confirmed<br>• A progressive increase in the number of 10 Fr MPSs at 3-month intervals was planned with an overall duration of treatment of 9 to 12 months according to the rate of improvement of the AS<br>• All stents were removed at each session<br>• Hydrostatic balloon dilatation to assist the placement of MPS across the AS was used when the number of stents had to be increased<br>• Maximum number of MPS was chosen according to the diameters of the native and donor CBDs	No industry sources
Coté, 2016	• Bismuth Type I BBS	• Suspected malignant etiology of the stricture	• Fully CSEMS (fully WallFlex, Boston Scientific) of	• Stricture was dilated to the safest maximum	• The National Institute of Diabetes and Digestive and

	• Objective signs/symptoms related to the stricture	• Prior endotherapy within one year of presentation except in Early (< 30 days) stent placement following LT, or in patients with CP, single plastic stent placed during presenting ERCP while evaluating for malignancy<br>• Bismuth Type II-IV stricture<br>• Proximal CHD diameter < 6 mm<br>• Intact gallbladder except in cases where a stent can be deployed > 1cm below the cystic duct insertion<br>• Age < 18 years<br>• Pregnancy<br>• Incarceration<br>• Inability to provide informed consent<br>• Karnofsky score ≤ 40<br>• Inability to pass a guidewire proximal to the stricture<br>• Stricture > 8cm in length<br>• Life expectancy < 1 year<br>• Concomitant non-AS (e.g., biliary cast syndrome)	adequate length to cross the stricture and the papilla were used<br>• If necessary, the stricture was dilated before the CSEMS was deployed<br>• To prevent CSEMS migration, an 8-mm diameter CSEMS was used for bile ducts measuring 6 to 7 mm, and a 10-mm diameter CSEMS for bile ducts 8 mm or larger<br>• Given the superior patency of CSEMSs compared to MPSs, a follow-up ERCP was performed 6 months after randomization<br>• If the stricture persisted at the repeat ERCP, the CSEMS was replaced for another 6-month period.	diameter based on the endoscopist's judgment<br>• Maximum cumulative diameter of MPSs was deployed<br>• A repeat ERCP was performed 3 to 4 months later, at which point all plastic stents were removed and the stricture was evaluated for resolution<br>• If the stricture remained, the cumulative diameter of the MPSs was increased as much as possible<br>• ERCPs were repeated every 3 to 4 months with necessary up-sizing of the MPSs until the stricture was resolved	Kidney Diseases of the National Institutes of Health (R21DK090708),<br>• An American Society for Gastrointestinal Endoscopy Career Development Award
Haapamäki, 2015	• Patients admitted for ERCP to treat BBS due to suspected biliary obstruction caused by CP with or without AP as judged by elevated BR and/or ALP	• Patients with malignancies<br>• Patients with known liver cirrhosis<br>• Patients with acute or chronic hepatitis	• At the initial ERCP, an endoscopic sphincterotomy was performed and a 10-Fr plastic stent was inserted to treat cholestasis<br>• CBD dilation was done only if necessary, and any	• At the initial ERCP, an endoscopic sphincterotomy was performed and a 10-Fr plastic stent was inserted to treat cholestasis<br>• CBD dilation was done only if necessary, and any	No industry sources

		• Patients with abnormal hepatic imaging studies<br>• Patients with their first attack of AP	existing CBD stones above the stricture were removed<br>• Pancreatic stents were inserted if required<br>• At the second ERCP, the stricture was dilated with an 8-mm balloon before stent placement<br>• The original plastic stent was replaced with a single CSEMS (diameter 10mm, length 6cm)<br>• At 3 months, the stent's position and function were checked, and if there was stent migration, it was replaced<br>• All stents were removed 6 months after randomization	existing CBD stones above the stricture were removed<br>• Pancreatic stents were inserted if required<br>• At the second ERCP, the stricture was dilated with an 8-mm balloon before stent placement<br>• The original plastic stent was replaced with three MPSs<br>• At 3 months, the number of MPSs was increased to a maximum of six 10-Fr stents if possible<br>• All stents were removed 6 months after randomization	
Kaffes, 2014	• Post-liver-transplant biliary ASs<br>• Age > 18 years<br>• Willing to take part in the trial<br>• Cadaveric LT	• Non-ASs<br>• Hepatic artery thrombosis<br>• Intrahepatic biliary disease<br>• All strictures on ERCP proximal to and involving the hilum<br>• Patients with leaks alone	• Once the AS was confirmed, a long 480 cm guide wire (metro tracer, Cook Medical, WinstonSalem, NC) was inserted across the stricture<br>• The fully CSEMS (Taewoong Medical, Seoul, Korea) was then placed into position<br>• The stent had a diameter of 10 mm at either end and had a gradual narrowing into the center to 8 mm diameter. The fixed stent length was 40 mm with a 10 cm suture<br>• At 12 weeks, the stents were removed to assess the stricture	• Once the AS was confirmed, the endoscopist then aimed for MPS insertion preferring a maximum stent number and a 10 Fr caliber (Cook Medical, Winston-Salem, NC)<br>• It was up to the discretion of the endoscopist to dilate<br>• Removal of the stents via ERCP and an assessment of the stricture was done in 3-monthly intervals<br>• If the stricture resolved prior to the 12-month period, the patient was not stented and placed on clinical follow up instead	No specific grant from any funding agency in the public, commercial, or not-for-profit sectors

			• If the stricture had resolved, no further stenting was performed, and patient was placed on clinical follow up.		
Martins, 2018	• Age 18-75 years<br>• Post-orthotopic LT AS Indication for endoscopic therapy<br>• The stricture should be located at least 2 cm below hepatic confluence	• Pregnancy<br>• Non-AS or hilar stricture,<br>• Isolated biliary fistulae,<br>• Hepatic artery stenosis/thrombosis,<br>• Refusal for randomization<br>• Orthotopic LT within 1 month of the endoscopic procedure	• A guidewire was passed through the AS<br>• The stent length was long enough to be 1 cm above the stricture and not more than 1 cm exteriorizing the papilla<br>• A CSEMS (Wallflex, Boston Scientific, 10 mm in diameter, 60 or 80 mm in length) was then advanced over the guidewire and deployed, with dilation and/or sphincterotomy performed if necessary<br>• Stent removal was scheduled after a 6-month indwelling period<br>• If there were signs of stent occlusion or migration, ERCP was performed earlier than scheduled	• A guidewire was passed through the AS<br>• Biliary sphincterotomy was performed to facilitate the placement of MPSs<br>• AS was dilated to the maximum safe diameter (6-10 mm) using a hydrostatic balloon, and the maximum number of MPSs that could fit within the stricture was deployed<br>• ERCP was repeated every 3 months, during which all MPSs were removed, the stricture was progressively dilated, and an increasing number of stents were placed at each session, continuing for 12 months<br>• If there were signs of stent occlusion or migration, ERCP was performed earlier than scheduled	No industry sources
Ramchandani, 2021	• Age ≥ 18 years<br>• Documented CP<br>• Symptomatic BBS documented at time of enrollment for naïve stricture or at the time of prior plastic stent placement in strictures that had 1 prior plastic stent inserted<br>• CBD stricture based on imaging assessment of	• Non-CP etiology of BBS<br>• Malignant bile duct stricture<br>• Prior biliary metal stent or more than 1 plastic stent of 10 Fr size or smaller for ≥ 6 months	• Single 8-mm or 10-mm- diameter fully CSEMS was deployed for a planned indwell time of 12 months<br>• At 12 months, stent was removed, and a cholangiogram or other CBD imaging and liver enzyme tests were performed	• 3 or 4 side-by-side MPS stenting procedures over a 12-month treatment period were performed<br>• At least two 8.5 or 10 Fr. MPSs were deployed whenever possible<br>• At 4 and 8 months, follow up visits for MPS exchange or increase the	Boston Scientific Corporation

	dilatation of the common and/or intrahepatic bile ducts	• Developing obstructive biliary symptoms associated with onset of AP<br>• Stricture within 2 cm of CBD bifurcation<br>• Known bile duct fistula or leak<br>• Symptomatic duodenal stenosis with gastric stasis<br>• Contraindication to endoscopic techniques or devices<br>• Participation in another investigational study within 90 days before consent<br>• Exclusion by investigator discretion	• At 24 months, assessments of biliary obstructive symptoms, adverse events, and liver enzyme tests were conducted	number of stents and liver enzyme tests were performed<br>• At 12 months, stents were removed, and a cholangiogram or other CBD imaging and liver enzyme tests were performed<br>• At 24 months, assessments of biliary obstructive symptoms, adverse events, and liver enzyme tests were conducted	
Tal, 2017	• Patients who, underwent deceased donor orthotopic LT for end-stage liver disease or acute liver failure<br>• AS suspected or confirmed by ERCP<br>• Did not participate in other studies	• Patients with non-AS, including ischemic-type biliary lesions or biliodigestive anastomosis<br>• MPSs or CSEMSs were judged technically not feasible or reasonable by the investigator	• After confirmation and grading of ASs, endoscopic sphincterotomy was performed<br>• Hydrostatic balloon dilatation before stent placement (eg, 6 or 8 mm MaxForce balloon catheter; Boston Scientific, Ratingen, Germany) was at the discretion of the investigator<br>• Three different fully CSEMS and without antimigration flaps with a diameter of 10 mm were used<br>• For retrieval after 4 to 6 months, 2 stent types had a small retrieval flap and 1 CSEMS had a big lasso. Two CSEMS were placed across the papilla and 1 CSEMS type was	• After confirmation and grading of ASs, endoscopic sphincterotomy was performed<br>• Hydrostatic balloon dilatation before stent placement (eg, 6 or 8 mm MaxForce balloon catheter; Boston Scientific, Ratingen, Germany) was at the discretion of the investigator<br>• As many MPSs as possible with the optimal diameter (eg, 7F, 10F, 11.5F) were deployed<br>• Every 6 to 12 weeks, stent exchange ERCPs were performed and the number and diameter of the MPSs were increased as considered appropriate	No industry sources

			placed completely inside the CBD	• 4 different plastic stent types were used: straight plastic stents with 2 flaps, plastic stents with a duodenal bend and 2 flaps, plastic stents with a central bend and 2 flaps, or double pigtail stents	

ALP: Alkaline phosphatase; AP: Acute pancreatitis; AS: Anastomotic stricture; BBS: Benign biliary stricture; BR: Bilirubin; CBD: Common bile duct; CHD: Common hepatic duct; CP: Chronic pancreatitis; CSEMS: Covered self-expandable metal stents; ERCP: Endoscopic retrograde cholangiopancreatography; LT: liver transplant; MPS: Multiple plastic stents; MR: Magnetic resonance; MRI: Magnetic resonance imaging

Fig. 1 Risk of bias assessment based on (RoB2 tool) barplot showing overall quality of studies across different domains

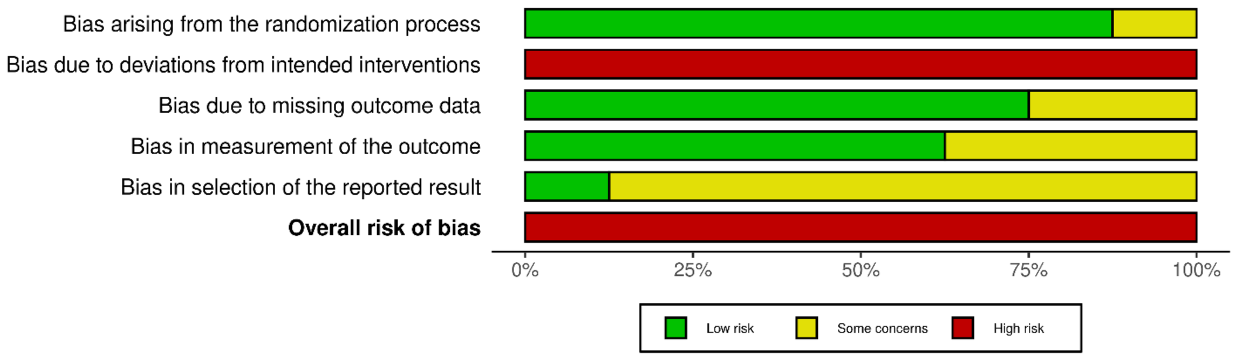

Fig. 2 Risk of bias assessment based on (RoB2 tool) traffic light showing quality of individual studies across different domains

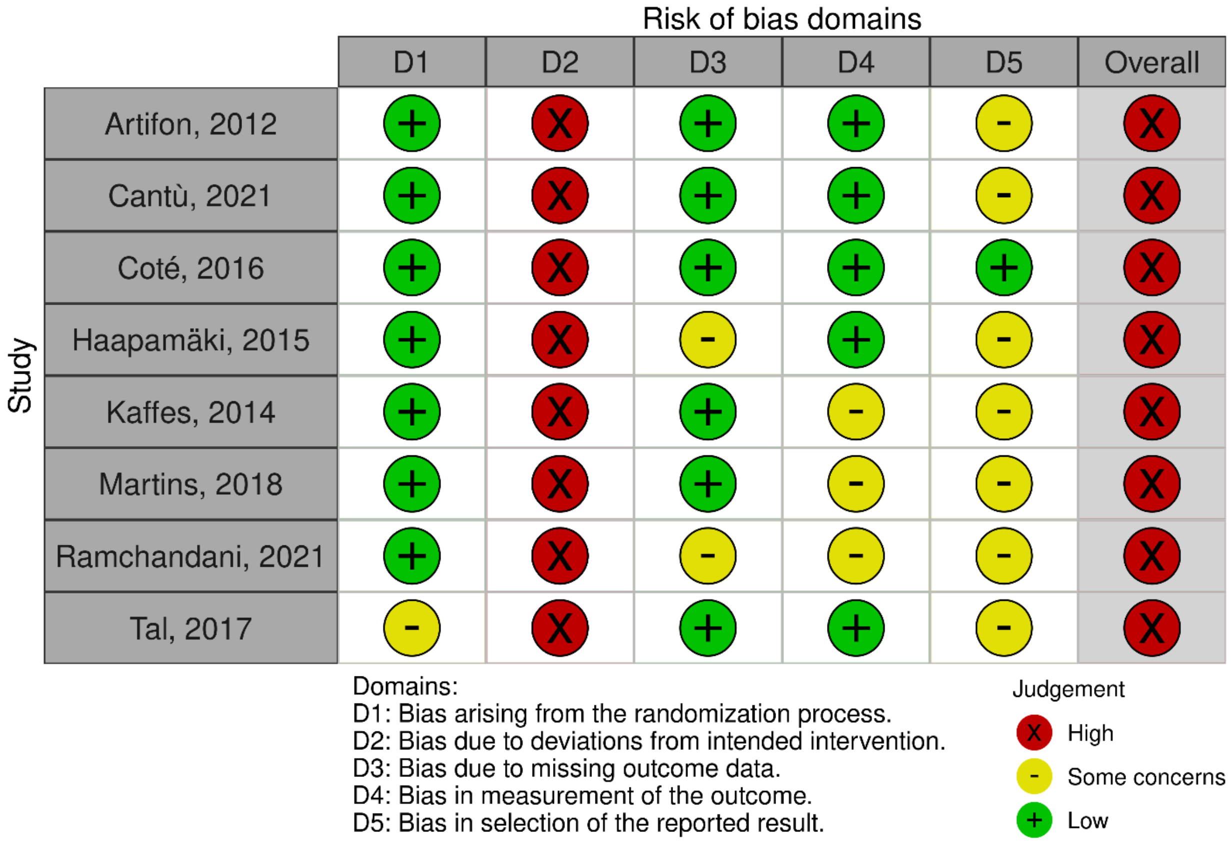

Table 3 Outcomes

Outcome	Analysis	Subgroup	Random or fixed effects	ITT or PP	No. of studies	No. of patients	Effect estimate (95% CI)	p-value	I^2
Stricture resolution	Overall	-	Random effects	ITT	7 RCTs	499	RR 1.04 (0.97 to 1.11)	0.24	9%
Stricture resolution	Overall	-	Fixed effects	ITT	7 RCTs	499	RR 1.01 (0.91 to 1.11)	0.86	9%
Stricture resolution	Subgroup	Post-surgical	Random effects	ITT	1 RCT	31	RR 1.07 (0.73 to 1.56)	0.74	-
Stricture resolution	Subgroup	Post-surgical	Fixed effects	ITT	1 RCT	31	RR 1.07 (0.73 to 1.56)	0.74	-
Stricture resolution	Subgroup	OLT	Random effects	ITT	4 RCTs	205	RR 1.04 (0.97 to 1.11)	0.31	3%
Stricture resolution	Subgroup	OLT	Fixed effects	ITT	4 RCTs	205	RR 1.01 (0.92 to 1.12)	0.81	3%
Stricture resolution	Subgroup	CP	Random effects	ITT	3 RCTs	259	RR 1.06 (0.75 to 1.51)	0.73	60%
Stricture resolution	Subgroup	CP	Fixed effects	ITT	3 RCTs	259	RR 1.00 (0.83 to 1.20)	0.98	60%
Stricture resolution	Overall	-	Random effects	ITT-best case scenario	7 RCTs	499	RR 1.14 (1.03 to 1.25)	< 0.01	37%
Stricture resolution	Overall	-	Fixed effects	ITT-best case scenario	7 RCTs	499	RR 1.18 (1.08 to 1.28)	< 0.01	37%
Stricture resolution	Overall	-	Random effects	ITT-worst case scenario	7 RCTs	499	RR 0.92 (0.80 to 1.05)	0.21	66%
Stricture resolution	Overall	-	Fixed effects	ITT-worst case scenario	7 RCTs	499	RR 0.87 (0.80 to 0.95)	< 0.01	66%
Stricture resolution	Overall	-	Random effects	PP	7 RCTs	429	RR 1.01 (0.95 to 1.08)	0.75	3%
Stricture resolution	Overall	-	Fixed effects	PP	7 RCTs	429	RR 1.01 (0.94 to 1.09)	0.75	3%
Stricture resolution	Subgroup	Post-surgical	Random effects	PP	1 RCT	31	RR 1.07 (0.73 to 1.56)	0.74	-
Stricture resolution	Subgroup	Post-surgical	Fixed effects	PP	1 RCT	31	RR 1.07 (0.73 to 1.56)	0.74	-
Stricture resolution	Subgroup	OLT	Random effects	PP	4 RCTs	190	RR 0.99 (0.91 to 1.09)	0.89	38%
Stricture resolution	Subgroup	OLT	Fixed effects	PP	4 RCTs	190	RR 0.99 (0.90 to 1.09)	0.84	38%
Stricture resolution	Subgroup	CP	Random effects	PP	3 RCTs	214	RR 1.04 (0.91 to 1.19)	0.55	50%
Stricture resolution	Subgroup	CP	Fixed effects	PP	3 RCTs	214	RR 1.08 (0.93 to 1.25)	0.31	50%
Stricture resolution after initial treatment	Overall	-	Random effects	ITT	2 RCTs	50	RR 0.84 (0.33 to 2.15)	0.72	75%
Stricture resolution	Overall	-	Fixed effects	ITT	2 RCTs	50	RR 0.78 (0.50 to 1.21)	0.27	75%

Outcome	Analysis	Subgroup	Random or fixed effects	ITT or PP	No. of studies	No. of patients	Effect estimate (95% CI)	p-value	I^2
after initial treatment									
Stricture recurrence	Overall	-	Random effects	ITT	5 RCTs	251	RR 1.27 (0.66 to 2.45)	0.47	31%
Stricture recurrence	Overall	-	Fixed effects	ITT	5 RCTs	251	RR 1.78 (0.97 to 3.27)	0.06	31%
Stricture recurrence	Subgroup	OLT	Random effects	ITT	4 RCTs	182	RR 1.80 (0.57 to 5.63)	0.31	48%
Stricture recurrence	Subgroup	OLT	Fixed effects	ITT	4 RCTs	182	RR 2.15 (1.08 to 4.27)	0.03	48%
Stricture recurrence	Subgroup	CP	Random effects	ITT	2 RCTs	65	RR 0.84 (0.22 to 3.23)	0.80	0%
Stricture recurrence	Subgroup	CP	Fixed effects	ITT	2 RCTs	65	RR 0.84 (0.22 to 3.20)	0.80	0%
Technical success	Overall	-	Random effects	ITT	3 RCTs	307	RR 1.00 (0.98 to 1.03)	0.73	0%
Technical success	Overall	-	Fixed effects	ITT	3 RCTs	307	RR 1.01 (0.98 to 1.03)	0.68	0%
Stent migration	Overall	-	Random effects	ITT	7 RCTs	499	IRR 1.13 (0.72 to 1.78)	0.61	12%
Stent migration	Overall	-	Fixed effects	ITT	7 RCTs	499	IRR 1.29 (0.83 to 2.00)	0.25	12%
Stent migration	Subgroup	Post-surgical	Random effects	ITT	1 RCT	31	IRR 5.33 (0.26 to 111.09)	0.28	-
Stent migration	Subgroup	Post-surgical	Fixed effects	ITT	1 RCT	31	IRR 5.33 (0.26 to 111.09)	0.28	-
Stent migration	Subgroup	Mixed etiologies	Random effects	ITT	1 RCT	112	IRR 1.54 (0.70 to 3.40)	0.28	-
Stent migration	Subgroup	Mixed etiologies	Fixed effects	ITT	1 RCT	112	IRR 1.54 (0.70 to 3.40)	0.28	-
Stent migration	Subgroup	CP	Random effects	ITT	2 RCTs	224	IRR 0.85 (0.45 to 1.60)	0.61	0%
Stent migration	Subgroup	CP	Fixed effects	ITT	2 RCTs	224	IRR 0.84 (0.45 to 1.60)	0.60	0%
Stent migration	Subgroup	OLT	Random effects	ITT	3 RCTs	132	IRR 1.49 (0.19 to 11.97)	0.71	54%
Stent migration	Subgroup	OLT	Fixed effects	ITT	3 RCTs	132	IRR 2.20 (0.76 to 6.33)	0.14	54%
All-cause mortality	Overall	-	Random effects	ITT	7 RCTs	501	RR 1.22 (0.41 to 3.62)	0.72	25%
All-cause mortality	Overall	-	Fixed effects	ITT	7 RCTs	501	RR 1.08 (0.51 to 2.32)	0.84	25%
All-cause mortality	Subgroup	Post-surgical	Random effects	ITT	1 RCT	31	Not estimable	-	-
All-cause mortality	Subgroup	Post-surgical	Fixed effects	ITT	1 RCT	31	Not estimable	-	-
All-cause mortality	Subgroup	Mixed etiologies	Random effects	ITT	1 RCT	112	RR 0.28 (0.06 to 1.27)	1.00	-
All-cause mortality	Subgroup	Mixed etiologies	Fixed effects	ITT	1 RCT	112	RR 0.28 (0.06 to 1.27)	1.00	-
All-cause mortality	Subgroup	CP	Random effects	ITT	2 RCTs	224	RR 2.75 (0.75 to 10.10)	0.13	0%

Outcome	Analysis	Subgroup	Random or fixed effects	ITT or PP	No. of studies	No. of patients	Effect estimate (95% CI)	p-value	I^2
All-cause mortality	Subgroup	CP	Fixed effects	ITT	2 RCTs	224	RR 2.75 (0.75 to 10.10)	0.13	0%
All-cause mortality	Subgroup	OLT	Random effects	ITT	3 RCTs	134	RR 1.49 (0.25 to 8.79)	0.66	0%
All-cause mortality	Subgroup	OLT	Fixed effects	ITT	3 RCTs	134	RR 1.50 (0.26 to 8.65)	0.65	0%
Adverse events	Overall	-	Random effects	ITT	7 RCTs	499	IRR 1.13 (0.86 to 1.47)	0.38	5%
Adverse events	Overall	-	Fixed effects	ITT	7 RCTs	499	IRR 1.12 (0.86 to 1.46)	0.40	5%
Adverse events	Subgroup	Post-surgical	Random effects	ITT	1 RCT	31	IRR 1.60 (0.45 to 5.67)	0.48	-
Adverse events	Subgroup	Post-surgical	Fixed effects	ITT	1 RCT	31	IRR 1.60 (0.45 to 5.67)	0.48	-
Adverse events	Subgroup	Mixed etiologies	Random effects	ITT	1 RCT	112	IRR 1.10 (0.70 to 1.70)	0.67	-
Adverse events	Subgroup	Mixed etiologies	Fixed effects	ITT	1 RCT	112	IRR 1.10 (0.70 to 1.70)	0.67	-
Adverse events	Subgroup	CP	Random effects	ITT	2 RCTs	224	IRR 1.04 (0.68 to 1.59)	0.86	0%
Adverse events	Subgroup	CP	Fixed effects	ITT	2 RCTs	224	IRR 1.04 (0.68 to 1.59)	0.86	0%
Adverse events	Subgroup	OLT	Random effects	ITT	3 RCTs	132	IRR 1.12 (0.24 to 5.17)	0.88	65%
Adverse events	Subgroup	OLT	Fixed effects	ITT	3 RCTs	132	IRR 1.24 (0.70 to 2.20)	0.47	65%
Stent treatment duration (days)	Overall	-	Random effects	ITT	5 RCTs	382	MD -98.62 (-180.80 to -16.43)	0.02	96%
Stent treatment duration (days)	Overall	-	Fixed effects	ITT	5 RCTs	382	MD -119.02 (-137.41 to -100.62)	< 0.01	96%
Stent treatment duration (days)	Subgroup	OLT	Random effects	ITT	4 RCTs	191	MD -121.63 (-209.09 to -34.17)	< 0.01	94%
Stent treatment duration (days)	Subgroup	OLT	Fixed effects	ITT	4 RCTs	191	MD -147.92 (-168.45 to -127.40)	< 0.01	94%
Stent treatment duration (days)	Subgroup	CP	Random effects	ITT	2 RCTs	187	MD -18.68 (-62.72 to 25.35)	0.41	44%
Stent treatment duration (days)	Subgroup	CP	Fixed effects	ITT	2 RCTs	187	MD -15.34 (-46.86 to 16.17)	0.34	44%
Number of ERCP procedures	Overall	-	Random effects	ITT	5 RCTs	382	MD -1.84 (-2.56 to -1.11)	< 0.01	76%

Outcome	Analysis	Subgroup	Random or fixed effects	ITT or PP	No. of studies	No. of patients	Effect estimate (95% CI)	p-value	I^2
Number of ERCP procedures	Overall	-	Fixed effects	ITT	5 RCTs	382	MD -1.39 (-1.64 to -1.14)	< 0.01	76%
Number of ERCP procedures	Subgroup	OLT	Random effects	ITT	4 RCTs	191	MD -1.99 (-2.97 to -1.01)	< 0.01	86%
Number of ERCP procedures	Subgroup	OLT	Fixed effects	ITT	4 RCTs	191	MD -1.29 (-1.60 to -0.99)	< 0.01	86%
Number of ERCP procedures	Subgroup	CP	Random effects	ITT	2 RCTs	187	MD -1.34 (-1.70 to -0.99)	< 0.01	0%
Number of ERCP procedures	Subgroup	CP	Fixed effects	ITT	2 RCTs	187	MD -1.34 (-1.70 to -0.99)	< 0.01	0%
Number of stents	Overall	-	Random effects	ITT	7 RCTs	492	MD -6.04 (-9.55 to -2.52)	< 0.01	93%
Number of stents	Overall	-	Fixed effects	ITT	7 RCTs	492	MD -4.42 (-5.20 to -3.64)	< 0.01	93%
Number of stents	Subgroup	Post-surgical	Random effects	ITT	1 RCT	31	MD -3.80 (-6.73 to -0.87)	0.01	-
Number of stents	Subgroup	Post-surgical	Fixed effects	ITT	1 RCT	31	MD -3.80 (-6.73 to -0.87)	0.01	-
Number of stents	Subgroup	Mixed etiologies	Random effects	ITT	1 RCT	112	MD -0.82 (-2.32 to 0.68)	0.29	-
Number of stents	Subgroup	Mixed etiologies	Fixed effects	ITT	1 RCT	112	MD -0.82 (-2.32 to 0.68)	0.29	-
Number of stents	Subgroup	CP	Random effects	ITT	2 RCTs	222	MD -4.44 (-5.54 to -3.33)	< 0.01	0%
Number of stents	Subgroup	CP	Fixed effects	ITT	2 RCTs	222	MD -4.44 (-5.54 to -3.33)	< 0.01	0%
Number of stents	Subgroup	OLT	Random effects	ITT	3 RCTs	127	MD -9.85 (-15.82 to -3.89)	< 0.01	90%
Number of stents	Subgroup	OLT	Fixed effects	ITT	3 RCTs	127	MD -10.93 (-12.91 to -8.95)	< 0.01	90%

CI: Confidence interval; CP: Chronic pancreatitis; ERCP: Endoscopic retrograde cholangiopancreatography; ITT: Intention-to-treat; IRR: Incidence rate ratio; MD: Mean difference; OLT: Orthotopic liver transplantation; PP: Per-protocol; RCT: Randomized controlled trial; RR: Risk ratio

Table 4 Trial Sequential Analysis

Outcomes	№ of participants (studies)	Effect estimate (95%CI)	Effect estimate (TSA-adjusted 95%CI)	P-value	Heterogeneity (I^2)	Diversity (D^2)	DARIS	Conclusion (Z-curve crosses benefit, harm or futility boundaries, or DARIS reached)
Stricture resolution	499 (7 RCTs)	RR 1.04 (0.97 to 1.11)	RR 1.03 (0.92 to 1.16)	0.44	13%	27%	537	No difference between CSEMS and MPS*
Stricture resolution after initial treatment	50 (2 RCTs)	RR 0.84 (0.33 to 2.15)	NE†	0.72	75%	76%	1220	More trials are needed
Stricture recurrence	251 (5 RCTs)	RR 1.27 (0.66 to 2.45)	NE†	0.47	31%	45%	13,124	More trials are needed
Technical success	307 (3 RCTs)	RR 1.00 (0.98 to 1.03)	RR 1.00 (0.92 to 1.09)	0.78	0%	0%	2,928	More trials are needed
Stent migration	499 (7 RCTs)	IRR 1.13 (0.72 to 1.78)	IRR 1.11 (0.13 to 9.55)	0.71	17%	37%	8,285	More trials are needed
All-cause mortality	501 (7 RCTs)	RR 1.22 (0.41 to 3.62)	NE†	0.77	0%	0%	16,945	More trials are needed
Adverse events	499 (7 RCTs)	IRR 1.13 (0.86 to 1.47)	IRR 1.13 (0.42 to 3.04)	0.31	23%	46%	2,382	More trials are needed
Stent treatment duration (days)	382 (5 RCTs)	MD -119.02 (-137.41 to -100.62)	MD -119.02 (-157.39 to -80.64)	< 0.0001	97%	99%	300	CSEMS is superior to MPS‡
Number of ERCP procedures	382 (5 RCTs)	MD -1.84 (-2.56 to -1.11)	MD -1.83 (-2.76 to -0.90)	< 0.0001	77%	84%	184	CSEMS is superior to MPS‡
Number of stents	492 (7 RCTs)	MD -6.04 (-9.55 to -2.52)	MD -5.25 (-8.60 to -1.90)	0.0003	93%	94%	403	CSEMS is superior to MPS‡

DARIS: Diversity-adjusted required information size; IRR: Incidence rate ratio; MD: Mean difference; NE: Not estimable; RR: Risk ratio. DARIS is based on type I error 0.9%, type II error 10%, for binary outcomes relative risk reduction of 25% (except for stricture resolution relative risk reduction of 20% corresponding to risk difference of 15%), and for continuous outcomes we used the variance estimated from the pooled results.

* Z-curve crosses futility boundary even though DARIS is not reached.

† Alpha- and beta- spending boundaries were not computed due to insufficient information available for interim analyses. Calculations are according to conventional methods.

‡ Z-curve crosses the benefit boundary and DARIS is reached.

Table 5 Adverse events

Author, Year	Intervention (No. of patients)	Adverse events n(%)
Artifon, 2012	PCSEMS (15)	Hemorrhage 3(20)<br>Perforation 1(7)<br>Stent migration 2(13)<br>Ulcer 0(0)
	MPS (16)	Hemorrhage 1(6)<br>Perforation 2(13)<br>Stent migration 0(0)<br>Ulcer 1(6)
Cantù, 2021	FCSEMS (15)	Cholangitis 2(13)<br>Haemobilia 0(0)<br>Mild pancreatitis 1(7)<br>Stent migration 5(33)
	MPS (15)	Cholangitis 2(13)<br>Haemobilia 3(20)<br>Mild pancreatitis 1(7)<br>Stent migration 2(13)
Coté, 2016	CSEMS (57)	Abdominal pain 8(14)<br>Anorexia 3(5)<br>Bile duct obstruction 1(2)<br>Cardiopulmonary 1(2)<br>Cholangitis 2(4)<br>Cirrhosis or end stage liver disease 1(2)<br>Development of unrelated cancer 0(0)<br>Jaundice 1(2)<br>Nausea 1(2)<br>Neck swelling 0(0)<br>Pleural effusion 0(0)<br>Portal vein thrombosis 1(2)<br>Post ERCP pancreatitis 3(5)<br>Pruritus 1(2)<br>Pseudoaneurysm 1(2)<br>Stent induced changes to the bile duct 2(4)<br>Stent induced stricture 0(0)<br>Stent migration 16(28)<br>Stroke 0(0)<br>Urosepsis 0(0)
	MPS (55)	Abdominal pain 9(16)<br>Anorexia 0(0)<br>Bile duct obstruction 1(2)<br>Cardiopulmonary 1(2)<br>Cholangitis 1(2)<br>Cirrhosis or end stage liver disease 1(2)<br>Development of unrelated cancer 1(2)<br>Jaundice 1(2)<br>Nausea 0(0)<br>Neck swelling 1(2)<br>Pleural effusion 1(2)<br>Portal vein thrombosis 0(0)

Author, Year	Intervention (No. of patients)	Adverse events n(%)
		Post ERCP pancreatitis 3(5)<br>Pruritus 0(0)<br>Pseudoaneurysm 0(0)<br>Stent induced changes to the bile duct 5(9)<br>Stent induced stricture 0(0)<br>Stent migration 10(18)<br>Stroke 1(2)<br>Urosepsis 1(2)
Haapamäki, 2015	CSEMS<br>(30)	Acute cholecystitis 1(3)<br>Cholangitis 4(13)<br>Duodenal obstruction 1(3)<br>Intestinal perforation 0(0)<br>Pseudocyst bleeding 1(3)<br>Pseudocyst infection 0(0)<br>Stent migration 2(7)
	MPS<br>(30)	Acute cholecystitis 1(3)<br>Cholangitis 2(7)<br>Duodenal obstruction 0(0)<br>Intestinal perforation 1(3)<br>Pseudocyst bleeding 0(0)<br>Pseudocyst infection 2(7)<br>Stent migration 3(10)
Kaffes, 2014	FCSEMS<br>(10)	Cholangitis 1(10)<br>Pain 0(0)<br>Stent migration 0(0)
	MPS<br>(10)	Cholangitis 4(40)<br>Pain 1(10)<br>Stent migration 1(10)
Martins, 2018	CSEMS<br>(30)	Abdominal pain 1(3)<br>Abdominal pain with hospitalization 4(13)<br>Acute pancreatitis 8(27)<br>Bacteremia 1(3)<br>Sphincterotomy bleeding 0(0)<br>Stent migration 3(10)<br>Stent occlusion 0(0)
	MPS<br>(29)	Abdominal pain 0(0)<br>Abdominal pain with hospitalization 1(3)<br>Acute pancreatitis 3(10)<br>Bacteremia 2(7)<br>Sphincterotomy bleeding 3(10)<br>Stent migration 4(14)<br>Stent occlusion 2(7)

Author, Year	Intervention (No. of patients)	Adverse events n(%)
Ramchandani, 2021	FCSEMS (80)	Abdominal pain 5(6)<br>Acute on chronic pancreatitis 2(3)<br>Bacterial blood infection 1(1)<br>Cholangitis/fever/jaundice 7(9)<br>Cholecystitis 3(4)<br>Duodenal edema 1(1)<br>Perforation of duodenum 0(0)<br>Post ERCP pancreatitis 2(3)<br>Post-sphincterotomy bleed 1(1)<br>Stent migration 15(19)
	MPS (84)	Abdominal pain 5(6)<br>Acute on chronic pancreatitis 0(0)<br>Cholangitis/fever/jaundice 9(11)<br>Cholecystitis 1(1)<br>Mobile cholelithiasis 1(1)<br>Perforation of duodenum 1(1)<br>Post ERCP pancreatitis 0(0)<br>Post-sphincterotomy bleed 0(0)<br>Recurrent cholestasis 1(1)<br>Stent migration 18(21)
Tal, 2017	CSEMS (24)	Bilioduodenal fistula 0(0)<br>Severe haemobilia 0(0)<br>Stent migration 8(33)
	MPS (24)	Bilioduodenal fistula 1(4)<br>Severe haemobilia 1(4)<br>Stent migration 0(0)

Fig. 3 Pooled estimate of stricture resolution – intention to treat analysis

Study	CSEMS Events	Total	MPS Events	Total	RR [95%-CI]	Weight (common)	Weight (random)
Artifon, 2012	12	15	12	16	1.07 [0.73; 1.56]	6.2%	2.8%
Coté, 2016	50	57	41	55	1.18 [0.98; 1.41]	22.2%	12.3%
Haapamäki, 2015	20	30	22	30	0.91 [0.65; 1.27]	11.7%	3.7%
Kaffes, 2014	10	10	8	10	1.24 [0.92; 1.66]	4.5%	4.8%
Martins, 2018	25	32	28	32	0.89 [0.71; 1.12]	14.9%	8.1%
Ramchandani, 2021	47	80	54	84	0.91 [0.72; 1.17]	28.0%	6.9%
Tal, 2017	24	24	23	24	1.04 [0.96; 1.13]	12.5%	61.4%
**Common effect model**	**188**	**248**	**188**	**251**	**1.01 [0.91; 1.11]**	**100.0%**	--
**Random effects model**					**1.04 [0.97; 1.11]**	--	**100.0%**

Heterogeneity: $I^2 = 9\%$, $\tau^2 = 0$, $p = 0.36$

Test for overall effect (common effect): $z = 0.17$ ($p = 0.86$)

Test for overall effect (random effects): $z = 1.18$ ($p = 0.24$)

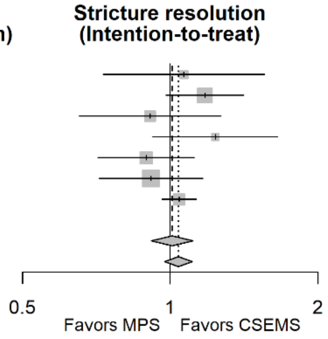

Fig. 4 Pooled estimate of stricture resolution – per protocol analysis

Study	CSEMS Events	Total	MPS Events	Total	RR [95%-CI]	Weight (common)	Weight (random)
Artifon, 2012	12	15	12	16	1.07 [0.73; 1.56]	6.4%	2.7%
Coté, 2016	50	54	41	48	1.08 [0.94; 1.25]	23.7%	20.5%
Haapamäki, 2015	20	22	22	25	1.03 [0.85; 1.26]	11.3%	10.3%
Kaffes, 2014	10	10	8	10	1.24 [0.92; 1.66]	4.6%	4.6%
Martins, 2018	25	30	28	29	0.86 [0.73; 1.03]	15.6%	13.0%
Ramchandani, 2021	47	62	54	70	0.98 [0.81; 1.19]	27.8%	11.0%
Tal, 2017	18	18	20	20	1.00 [0.90; 1.11]	10.7%	37.9%
**Common effect model**	**182**	**211**	**185**	**218**	**1.01 [0.94; 1.09]**	**100.0%**	**--**
**Random effects model**					**1.01 [0.95; 1.08]**	**--**	**100.0%**

Heterogeneity: $I^2 = 3\%$, $\tau^2 = 0$, $p = 0.40$
Test for overall effect (common effect): $z = 0.31$ ($p = 0.75$)
Test for overall effect (random effects): $z = 0.32$ ($p = 0.75$)

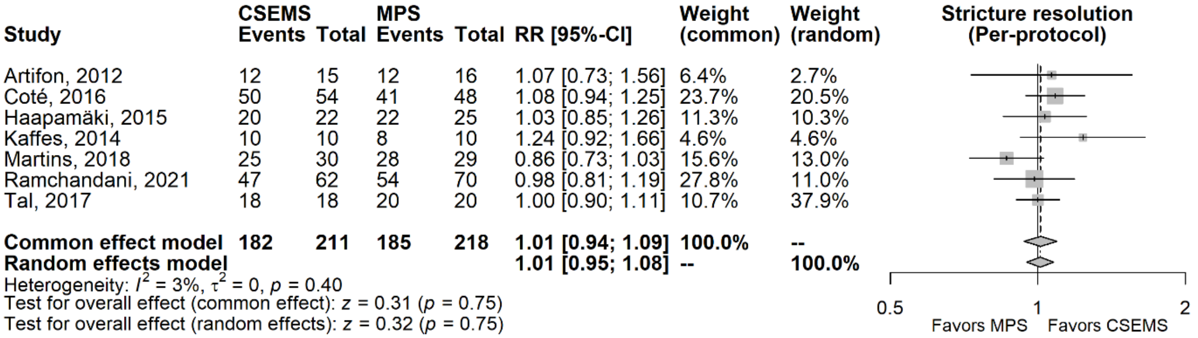

Fig. 5 Pooled estimate of stricture resolution – best case scenario

Study	CSEMS Events	CSEMS Total	MPS Events	MPS Total	RR [95%-CI]	Weight (common)	Weight (random)
Artifon, 2012	12	15	12	16	1.07 [0.73; 1.56]	6.2%	5.3%
Coté, 2016	53	57	41	55	1.25 [1.05; 1.48]	22.2%	16.9%
Haapamäki, 2015	28	30	22	30	1.27 [1.01; 1.61]	11.7%	11.2%
Kaffes, 2014	10	10	8	10	1.24 [0.92; 1.66]	4.5%	8.1%
Martins, 2018	27	32	28	32	0.96 [0.79; 1.18]	14.9%	14.1%
Ramchandani, 2021	65	80	54	84	1.26 [1.04; 1.53]	28.0%	14.8%
Tal, 2017	24	24	23	24	1.04 [0.96; 1.13]	12.5%	29.5%
**Common effect model**	**219**	**248**	**188**	**251**	**1.18 [1.08; 1.28]**	**100.0%**	**--**
**Random effects model**					**1.14 [1.03; 1.25]**	**--**	**100.0%**

Heterogeneity: $I^2 = 37\%$, $\tau^2 = 0.0061$, $p = 0.14$
Test for overall effect (common effect): $z = 3.77$ ($p < 0.01$)
Test for overall effect (random effects): $z = 2.64$ ($p < 0.01$)

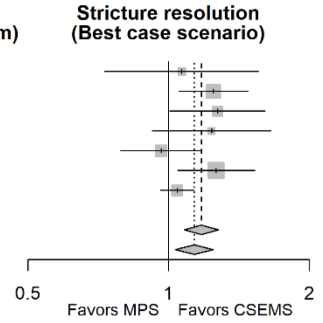

Fig. 6 Pooled estimate of stricture resolution – worst case scenario

Study	CSEMS Events	CSEMS Total	MPS Events	MPS Total	RR [95%-CI]	Weight (common)	Weight (random)
Artifon, 2012	12	15	12	16	1.07 [0.73; 1.56]	5.3%	8.1%
Coté, 2016	50	57	48	55	1.01 [0.87; 1.16]	22.4%	18.3%
Haapamäki, 2015	20	30	27	30	0.74 [0.56; 0.98]	12.4%	11.5%
Kaffes, 2014	10	10	8	10	1.24 [0.92; 1.66]	3.9%	11.0%
Martins, 2018	25	32	31	32	0.81 [0.66; 0.98]	14.2%	15.5%
Ramchandani, 2021	47	80	68	84	0.73 [0.59; 0.90]	30.5%	14.6%
Tal, 2017	24	24	24	24	1.00 [0.92; 1.08]	11.2%	21.0%
**Common effect model**	**188**	**248**	**218**	**251**	**0.87 [0.80; 0.95]**	**100.0%**	**--**
**Random effects model**					**0.92 [0.80; 1.05]**	**--**	**100.0%**

Heterogeneity: $I^2 = 66\%$, $\tau^2 = 0.0208$, $p < 0.01$
Test for overall effect (common effect): $z = -3.21$ ($p < 0.01$)
Test for overall effect (random effects): $z = -1.24$ ($p = 0.21$)

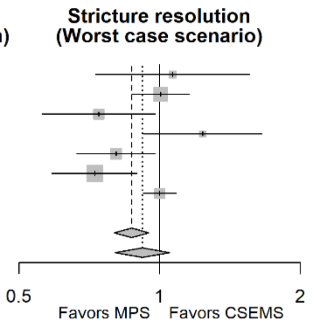

Fig. 7 Pooled estimate of stricture resolution – subgroup meta-analysis based on etiology- intention to treat analysis

Study	CSEMS Events	Total	MPS Events	Total	RR [95%-CI]	Weight (common)	Weight (random)
**Postsurgical strictures (cholecystectomies)**							
Artifon, 2012	12	15	12	16	1.07 [0.73; 1.56]	100.0%	100.0%
**Orthotopic Liver Transplant**							
Coté, 2016	33	37	31	36	1.04 [0.87; 1.23]	34.4%	15.7%
Kaffes, 2014	10	10	8	10	1.24 [0.92; 1.66]	9.3%	5.4%
Martins, 2018	25	32	28	32	0.89 [0.71; 1.12]	30.6%	9.2%
Tal, 2017	24	24	23	24	1.04 [0.96; 1.13]	25.7%	69.7%
**Common effect model**	**92**	**103**	**90**	**102**	**1.01 [0.92; 1.12]**	**100.0%**	**--**
**Random effects model**					**1.04 [0.97; 1.11]**	**--**	**100.0%**
Heterogeneity: I^2 = 3%, τ^2 = < 0.0001, p = 0.38							
Test for effect in subgroup (common effect): z = 0.24 (p = 0.81)							
Test for effect in subgroup (random effects): z = 1.02 (p = 0.31)							
**Chronic Pancreatitis**							
Coté, 2016	15	18	8	17	1.77 [1.03; 3.05]	9.9%	11.5%
Haapamäki, 2015	20	30	22	30	0.91 [0.65; 1.27]	26.5%	30.8%
Ramchandani, 2021	47	80	54	84	0.91 [0.72; 1.17]	63.5%	57.7%
**Common effect model**	**82**	**128**	**84**	**131**	**1.00 [0.83; 1.20]**	**100.0%**	**--**
**Random effects model**					**1.06 [0.75; 1.51]**	**--**	**100.0%**
Heterogeneity: I^2 = 60%, τ^2 = 0.0620, p = 0.08							
Test for effect in subgroup (common effect): z = -0.02 (p = 0.98)							
Test for effect in subgroup (random effects): z = 0.34 (p = 0.73)							

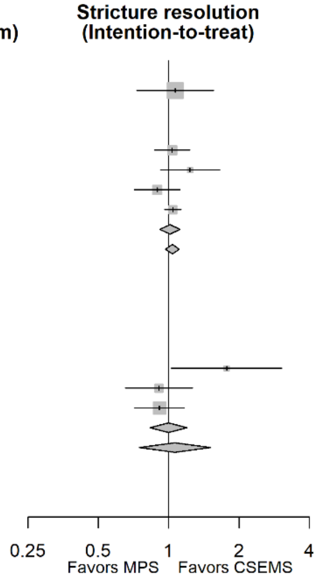

Test for subgroup differences (common effect): χ^2_2 = 0.10, df = 2 (p = 0.95)
Test for subgroup differences (random effects): χ^2_2 = 0.04, df = 2 (p = 0.98)

Fig. 8 Pooled estimate of stricture resolution – subgroup meta-analysis based on etiology – per protocol analysis

Study	CSEMS Events	Total	MPS Events	Total	RR [95%-CI]	Weight (common)	Weight (random)
Postsurgical strictures (cholecystectomies)							
Artifon, 2012	12	15	12	16	1.07 [0.73; 1.56]	100.0%	100.0%
Orthotopic Liver Transplant							
Coté, 2016	33	37	31	36	1.04 [0.87; 1.23]	35.8%	19.3%
Kaffes, 2014	10	10	8	10	1.24 [0.92; 1.66]	9.7%	6.7%
Martins, 2018	25	30	28	29	0.86 [0.73; 1.03]	32.4%	19.0%
Tal, 2017	18	18	20	20	1.00 [0.90; 1.11]	22.2%	55.0%
**Common effect model**	**86**	**95**	**87**	**95**	**0.99 [0.90; 1.09]**	**100.0%**	**--**
**Random effects model**					**0.99 [0.91; 1.09]**	**--**	**100.0%**
Heterogeneity: $I^2 = 38\%$, $\tau^2 = 0.0016$, $p = 0.18$							
Test for effect in subgroup (common effect): $z = -0.20$ ($p = 0.84$)							
Test for effect in subgroup (random effects): $z = -0.13$ ($p = 0.89$)							
Chronic Pancreatitis							
Coté, 2016	15	18	8	17	1.77 [1.03; 3.05]	10.3%	5.9%
Haapamäki, 2015	20	22	22	25	1.03 [0.85; 1.26]	25.9%	45.5%
Ramchandani, 2021	47	62	54	70	0.98 [0.81; 1.19]	63.8%	48.6%
**Common effect model**	**82**	**102**	**84**	**112**	**1.08 [0.93; 1.25]**	**100.0%**	**--**
**Random effects model**					**1.04 [0.91; 1.19]**	**--**	**100.0%**
Heterogeneity: $I^2 = 50\%$, $\tau^2 = < 0.0001$, $p = 0.13$							
Test for effect in subgroup (common effect): $z = 1.00$ ($p = 0.31$)							
Test for effect in subgroup (random effects): $z = 0.59$ ($p = 0.55$)							

Test for subgroup differences (common effect): $\chi^2_2 = 0.97$, df = 2 ($p = 0.62$)
Test for subgroup differences (random effects): $\chi^2_2 = 0.40$, df = 2 ($p = 0.82$)

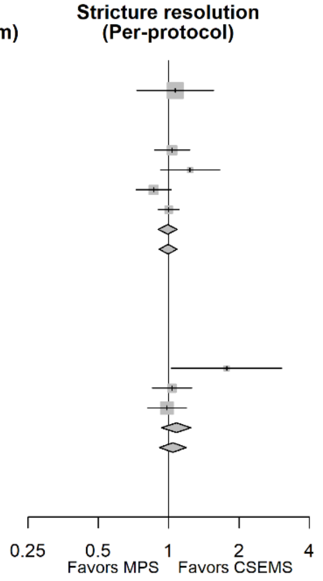

Fig. 9 Trial sequential analysis of stricture resolution

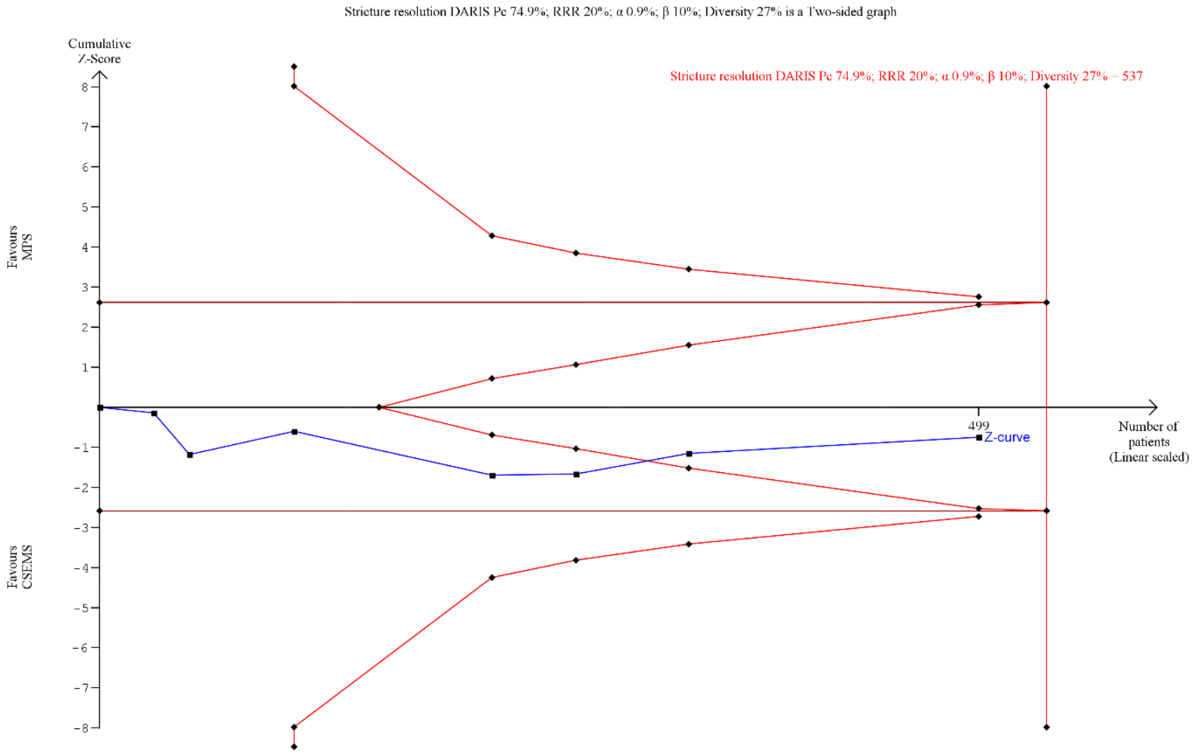

Fig. 10 Pooled estimate of stricture resolution after initial treatment

Study	CSEMS Events	Total	MPS Events	Total	RR [95%-CI]	Weight (common)	Weight (random)
Cantù, 2021	7	15	13	15	0.54 [0.30; 0.96]	72.2%	53.1%
Kaffes, 2014	7	10	5	10	1.40 [0.67; 2.94]	27.8%	46.9%
**Common effect model**	**14**	**25**	**18**	**25**	**0.78 [0.50; 1.21]**	**100.0%**	**--**
**Random effects model**					**0.84 [0.33; 2.15]**	**--**	**100.0%**

Heterogeneity: $I^2 = 75\%$, $\tau^2 = 0.3418$, $p = 0.05$
Test for overall effect (common effect): $z = -1.11$ ($p = 0.27$)
Test for overall effect (random effects): $z = -0.36$ ($p = 0.72$)

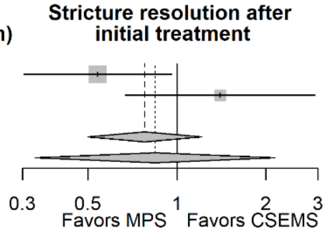

Fig. 11 Pooled estimate of technical success

Study	CSEMS Events	Total	MPS Events	Total	RR [95%-CI]	Weight (common)	Weight (random)
Artifon, 2012	15	15	16	16	1.00 [0.88; 1.13]	10.5%	4.4%
Coté, 2016	57	57	55	55	1.00 [0.97; 1.04]	37.0%	56.1%
Ramchandani, 2021	79	80	82	84	1.01 [0.97; 1.05]	52.5%	39.5%
**Common effect model**	**151**	**152**	**153**	**155**	**1.01 [0.98; 1.03]**	**100.0%**	**--**
**Random effects model**					**1.00 [0.98; 1.03]**	**--**	**100.0%**

Heterogeneity: $I^2 = 0\%$, $\tau^2 = 0$, $p = 0.91$
Test for overall effect (common effect): $z = 0.41$ ($p = 0.68$)
Test for overall effect (random effects): $z = 0.34$ ($p = 0.73$)

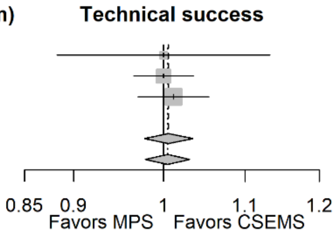

Fig. 12 Trial sequential analysis of technical success

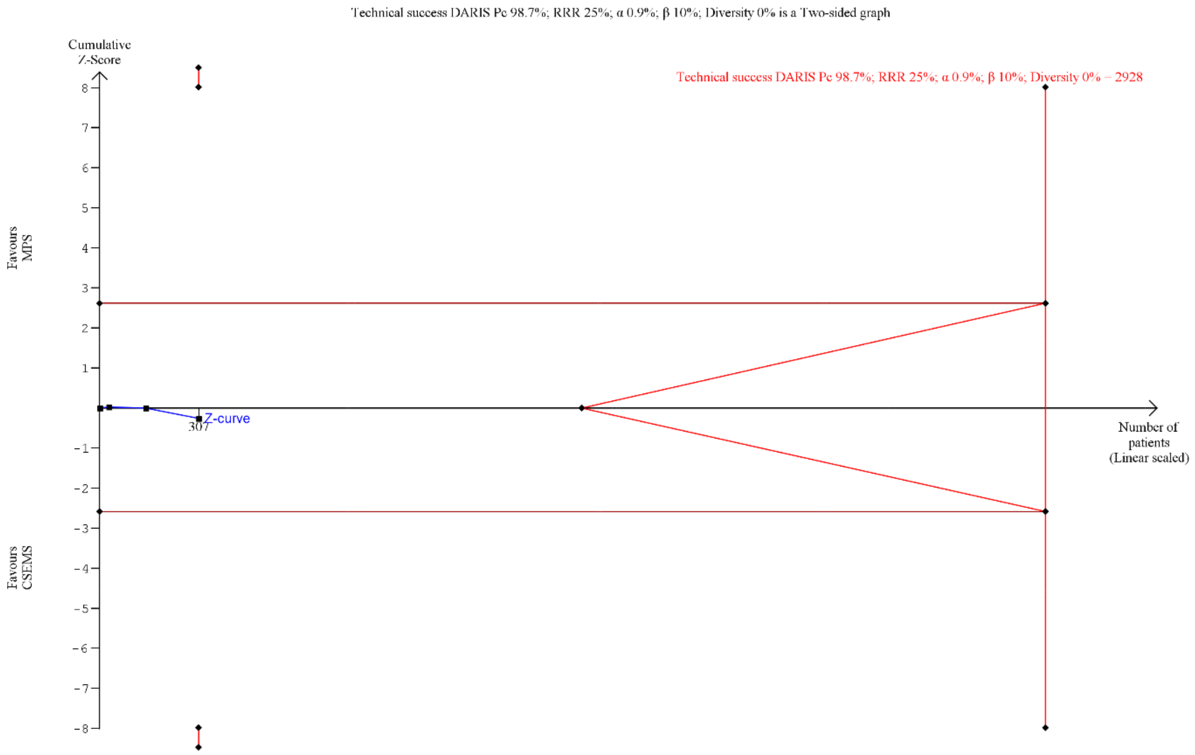

Fig. 13 Pooled estimate of stricture recurrence

Study	CSEMS Events	CSEMS Total	MPS Events	MPS Total	RR [95%-CI]	Weight (common)	Weight (random)
Coté, 2016	7	50	2	41	2.87 [0.63; 13.07]	15.7%	18.7%
Haapamäki, 2015	2	20	3	22	0.73 [0.14; 3.95]	20.5%	15.1%
Kaffes, 2014	3	10	3	8	0.80 [0.22; 2.94]	23.9%	25.3%
Martins, 2018	8	25	0	28	19.00 [1.15; 312.97]	3.4%	5.5%
Tal, 2017	5	24	5	23	0.96 [0.32; 2.88]	36.6%	35.5%
**Common effect model**	**25**	**129**	**13**	**122**	**1.78 [0.97; 3.27]**	**100.0%**	**--**
**Random effects model**					**1.27 [0.66; 2.45]**	**--**	**100.0%**

Heterogeneity: $I^2 = 31\%$, $\tau^2 < 0.0001$, $p = 0.21$
Test for overall effect (common effect): $z = 1.88$ ($p = 0.06$)
Test for overall effect (random effects): $z = 0.72$ ($p = 0.47$)

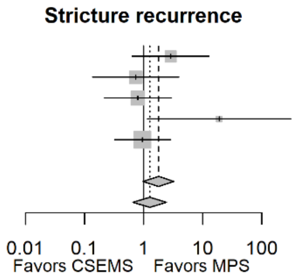

Fig. 14 Pooled estimate of stricture recurrence – subgroup analysis based on etiology

Study	CSEMS Events	CSEMS Total	MPS Events	MPS Total	RR [95%-CI]	Weight (common)	Weight (random)
**Orthotopic Liver Transplant**							
Coté, 2016	5	33	1	31	4.70 [0.58; 37.99]	10.4%	12.9%
Kaffes, 2014	3	10	3	8	0.80 [0.22; 2.94]	33.5%	33.3%
Martins, 2018	8	25	0	28	19.00 [1.15; 312.97]	4.8%	7.2%
Tal, 2017	5	24	5	23	0.96 [0.32; 2.88]	51.4%	46.6%
**Common effect model**	**21**	**92**	**9**	**90**	**2.15 [1.08; 4.27]**	**100.0%**	**--**
**Random effects model**					**1.80 [0.57; 5.63]**	**--**	**100.0%**
Heterogeneity: $I^2 = 48\%$, $\tau^2 = 0.6235$, $p = 0.12$							
Test for effect in subgroup (common effect): $z = 2.18$ ($p = 0.03$)							
Test for effect in subgroup (random effects): $z = 1.01$ ($p = 0.31$)							
**Chronic Pancreatitis**							
Coté, 2016	2	15	1	8	1.07 [0.11; 10.04]	31.3%	36.1%
Haapamäki, 2015	2	20	3	22	0.73 [0.14; 3.95]	68.7%	63.9%
**Common effect model**	**4**	**35**	**4**	**30**	**0.84 [0.22; 3.20]**	**100.0%**	**--**
**Random effects model**					**0.84 [0.22; 3.23]**	**--**	**100.0%**
Heterogeneity: $I^2 = 0\%$, $\tau^2 = 0$, $p = 0.79$							
Test for effect in subgroup (common effect): $z = -0.26$ ($p = 0.80$)							
Test for effect in subgroup (random effects): $z = -0.25$ ($p = 0.80$)							

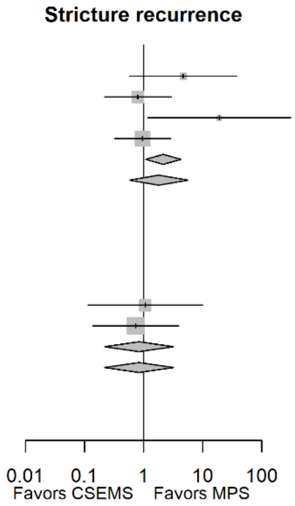

Test for subgroup differences (common effect): $\chi^2_1 = 1.50$, df = 1 ($p = 0.22$)
Test for subgroup differences (random effects): $\chi^2_1 = 0.71$, df = 1 ($p = 0.40$)

Fig. 15 Pooled estimate of adverse events - subgroup meta-analysis based on etiology

Study	CSEMS Events	CSEMS Total	MPS Events	MPS Total	IRR [95%-CI]	Weight (common)	Weight (random)
Postsurgical strictures (cholecystectomies)							
Artifon, 2012	6	15	4	16	1.60 [0.45; 5.67]	3.7%	4.5%
Mixed							
Coté, 2016	42	57	37	55	1.10 [0.70; 1.70]	36.0%	36.8%
Chronic Pancreatitis							
Haapamäki, 2015	9	30	9	30	1.00 [0.40; 2.52]	8.6%	8.4%
Ramchandani, 2021	34	80	34	84	1.05 [0.65; 1.69]	31.7%	31.8%
**Common effect model**	**43**	**110**	**43**	**114**	**1.04 [0.68; 1.59]**	**40.3%**	**--**
**Random effects model**					**1.04 [0.68; 1.59]**	**--**	**40.3%**
Heterogeneity: $I^2 = 0\%$, $\tau^2 = 0$, $p = 0.93$							
Test for effect in subgroup (common effect): $z = 0.18$ ($p = 0.86$)							
Test for effect in subgroup (random effects): $z = 0.18$ ($p = 0.86$)							
Orthotopic Liver Transplant							
Kaffes, 2014	1	10	6	10	0.17 [0.02; 1.38]	5.7%	1.6%
Martins, 2018	17	32	13	32	1.31 [0.64; 2.69]	12.4%	13.8%
Tal, 2017	8	24	2	24	4.00 [0.85; 18.84]	1.9%	3.0%
**Common effect model**	**26**	**66**	**21**	**66**	**1.24 [0.70; 2.20]**	**20.1%**	**--**
**Random effects model**					**1.12 [0.24; 5.17]**	**--**	**18.4%**
Heterogeneity: $I^2 = 65\%$, $\tau^2 = 1.2775$, $p = 0.06$							
Test for effect in subgroup (common effect): $z = 0.73$ ($p = 0.47$)							
Test for effect in subgroup (random effects): $z = 0.14$ ($p = 0.88$)							
**Common effect model**	**117**	**248**	**105**	**251**	**1.12 [0.86; 1.46]**	**100.0%**	**--**
**Random effects model**					**1.13 [0.86; 1.47]**	**--**	**100.0%**

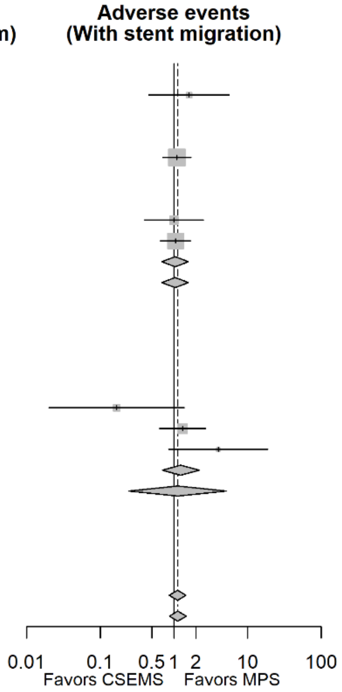

Heterogeneity: $I^2 = 5\%$, $\tau^2 < 0.0001$, $p = 0.39$
Test for overall effect (common effect): $z = 0.84$ ($p = 0.40$)
Test for overall effect (random effects): $z = 0.88$ ($p = 0.38$)
Test for subgroup differences (common effect): $\chi^2_3 = 0.55$, df = 3 ($p = 0.91$)
Test for subgroup differences (random effects): $\chi^2_3 = 0.40$, df = 3 ($p = 0.94$)

Fig. 16 Trial sequential analysis of adverse events

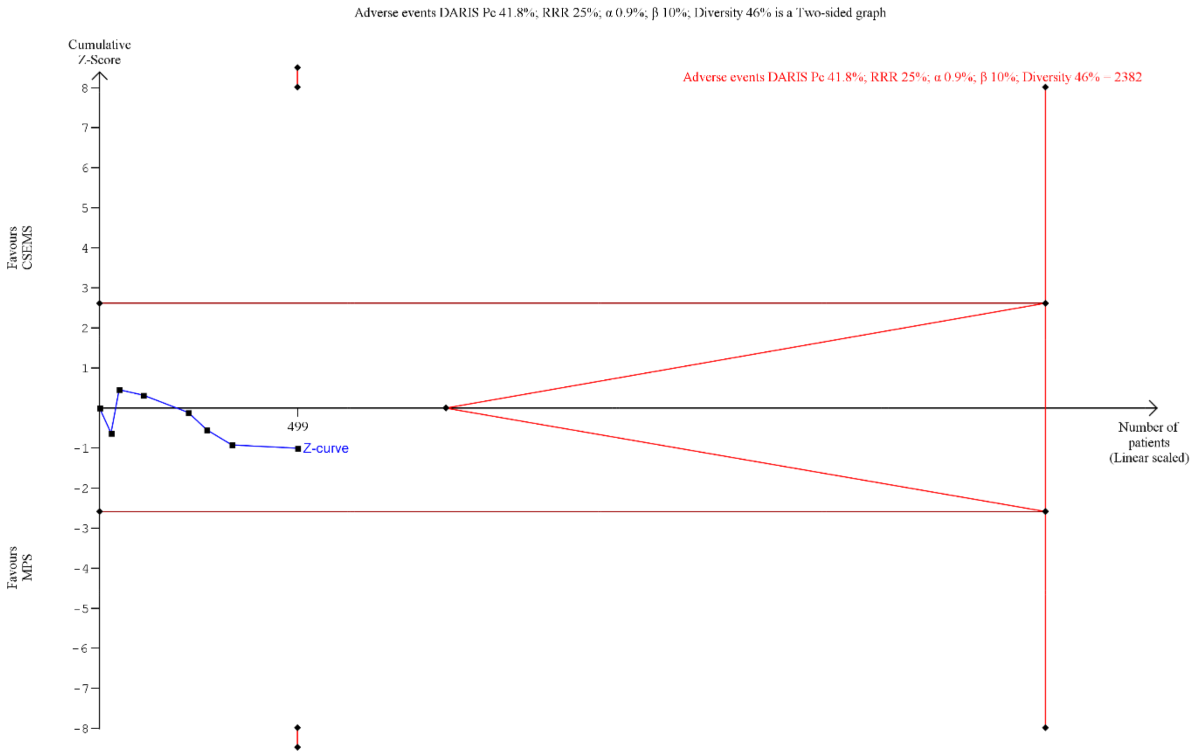

Fig. 17 Pooled estimate of all-cause mortality

Study	CSEMS Events	CSEMS Total	MPS Events	MPS Total	RR [95%-CI]	Weight (common)	Weight (random)
**Postsurgical strictures (cholecystectomies)**							
Artifon, 2012	0	15	0	16		0.0%	0.0%
**Mixed**							
Coté, 2016	2	57	7	55	0.28 [0.06; 1.27]	59.0%	27.8%
**Chronic Pancreatitis**							
Haapamäki, 2015	3	30	1	30	3.00 [0.33; 27.23]	8.3%	17.4%
Ramchandani, 2021	5	80	2	84	2.62 [0.52; 13.15]	16.2%	26.2%
**Common effect model**	**8**	**110**	**3**	**114**	**2.75 [0.75; 10.10]**	**24.4%**	**--**
**Random effects model**					**2.75 [0.75; 10.10]**	**--**	**43.6%**
Heterogeneity: $I^2 = 0\%$, $\tau^2 = 0$, $p = 0.92$							
Test for effect in subgroup (common effect): $z = 1.53$ ($p = 0.13$)							
Test for effect in subgroup (random effects): $z = 1.52$ ($p = 0.13$)							
**Orthotopic Liver Transplant**							
Kaffes, 2014	0	10	0	10		0.0%	0.0%
Martins, 2018	2	32	1	32	2.00 [0.19; 20.97]	8.3%	15.9%
Tal, 2017	1	25	1	25	1.00 [0.07; 15.12]	8.3%	12.7%
**Common effect model**	**3**	**67**	**2**	**67**	**1.50 [0.26; 8.65]**	**16.6%**	**--**
**Random effects model**					**1.49 [0.25; 8.79]**	**--**	**28.6%**
Heterogeneity: $I^2 = 0\%$, $\tau^2 = 0$, $p = 0.71$							
Test for effect in subgroup (common effect): $z = 0.45$ ($p = 0.65$)							
Test for effect in subgroup (random effects): $z = 0.44$ ($p = 0.66$)							
**Common effect model**	**13**	**249**	**12**	**252**	**1.08 [0.51; 2.32]**	**100.0%**	**--**
**Random effects model**					**1.22 [0.41; 3.62]**	**--**	**100.0%**

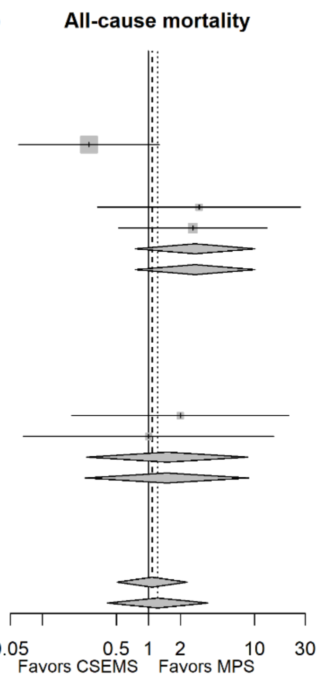

Heterogeneity: $I^2 = 25\%$, $\tau^2 = 0.5066$, $p = 0.26$
Test for overall effect (common effect): $z = 0.21$ ($p = 0.84$)
Test for overall effect (random effects): $z = 0.35$ ($p = 0.72$)
Test for subgroup differences (common effect): $\chi^2_2 = 5.18$, df = 2 ($p = 0.08$)
Test for subgroup differences (random effects): $\chi^2_2 = 5.16$, df = 2 ($p = 0.08$)

Fig. 18 Pooled estimate of stent migration

Study	CSEMS Events	CSEMS Total	MPS Events	MPS Total	IRR [95%-CI]	Weight (common)	Weight (random)
**Postsurgical strictures (cholecystectomies)**							
Artifon, 2012	2	15	0	16	5.33 [0.26; 111.09]	0.0%	2.3%
**Mixed**							
Coté, 2016	16	57	10	55	1.54 [0.70; 3.40]	28.5%	33.2%
**Chronic Pancreatitis**							
Haapamäki, 2015	2	30	3	30	0.67 [0.11; 3.99]	8.4%	6.5%
Ramchandani, 2021	15	80	18	84	0.88 [0.44; 1.74]	49.1%	44.2%
**Common effect model**	**17**	**110**	**21**	**114**	**0.84 [0.45; 1.60]**	**57.5%**	**--**
**Random effects model**					**0.85 [0.45; 1.60]**	**--**	**50.7%**
Heterogeneity: $I^2 = 0\%$, $\tau^2 = 0$, $p = 0.78$							
Test for effect in subgroup (common effect): $z = -0.52$ ($p = 0.60$)							
Test for effect in subgroup (random effects): $z = -0.52$ ($p = 0.61$)							
**Orthotopic Liver Transplant**							
Kaffes, 2014	0	10	1	10	0.33 [0.01; 8.18]	2.8%	2.0%
Martins, 2018	3	32	4	32	0.75 [0.17; 3.35]	11.2%	9.3%
Tal, 2017	8	24	0	24	17.00 [0.98; 294.53]	0.0%	2.6%
**Common effect model**	**11**	**66**	**5**	**66**	**2.20 [0.76; 6.33]**	**14.0%**	**--**
**Random effects model**					**1.49 [0.19; 11.97]**	**--**	**13.8%**
Heterogeneity: $I^2 = 54\%$, $\tau^2 = 1.8483$, $p = 0.12$							
Test for effect in subgroup (common effect): $z = 1.46$ ($p = 0.14$)							
Test for effect in subgroup (random effects): $z = 0.37$ ($p = 0.71$)							
**Common effect model**	**46**	**248**	**36**	**251**	**1.29 [0.83; 2.00]**	**100.0%**	**--**
**Random effects model**					**1.13 [0.72; 1.78]**	**--**	**100.0%**

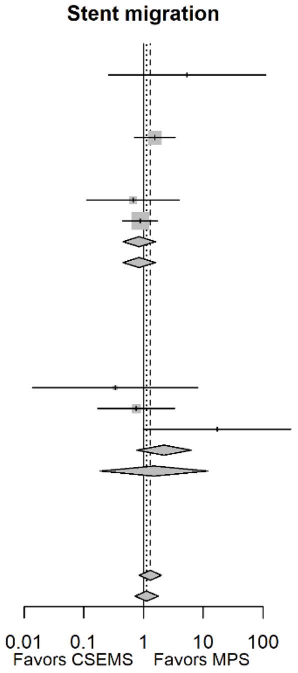

Heterogeneity: $I^2 = 12\%$, $\tau^2 < 0.0001$, $p = 0.34$
Test for overall effect (common effect): $z = 1.15$ ($p = 0.25$)
Test for overall effect (random effects): $z = 0.52$ ($p = 0.61$)
Test for subgroup differences (common effect): $\chi^2_3 = 2.81$, df = 3 ($p = 0.42$)
Test for subgroup differences (random effects): $\chi^2_3 = 2.46$, df = 3 ($p = 0.48$)

Fig. 19 Trial sequential analysis of stent migration

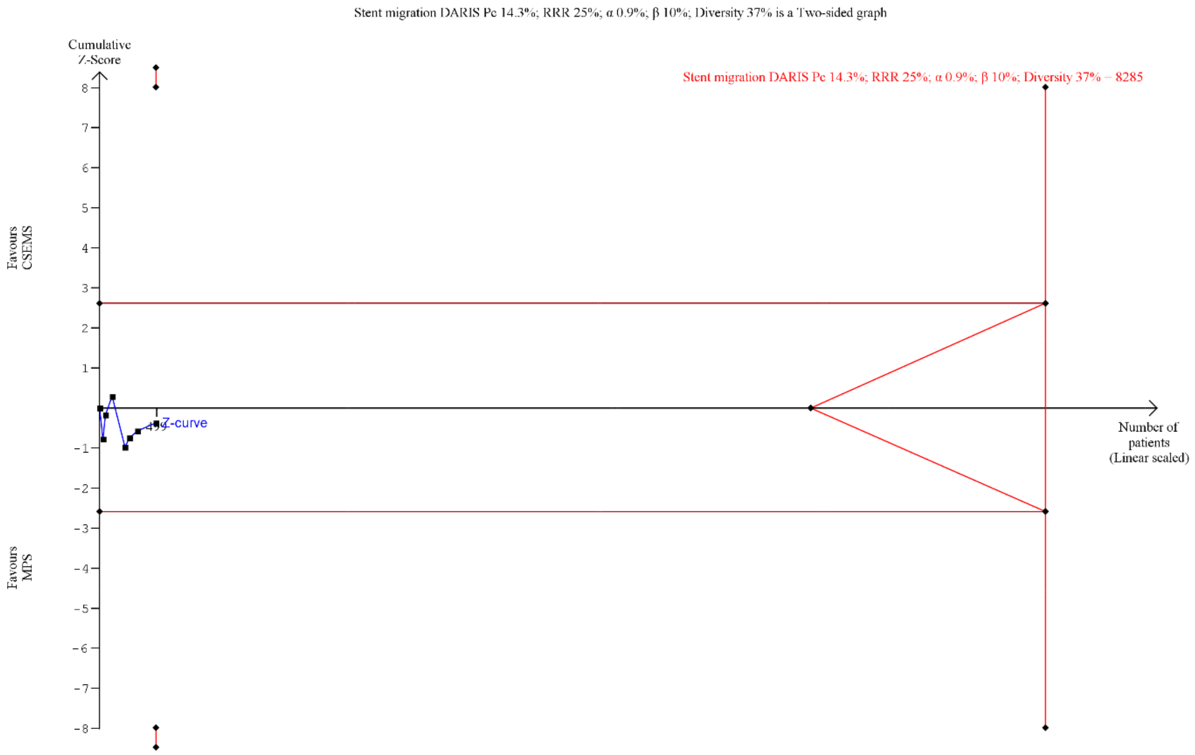

Fig. 20 Pooled estimate of number of ERCP

Study	Total (CSEMS)	Total (MPS)	MD [95%-CI]	Weight (common)	Weight (random)
Coté, 2016	50	41	-1.10 [-1.46; -0.74]	47.2%	26.0%
Kaffes, 2014	10	10	-2.50 [-3.75; -1.25]	3.9%	15.2%
Martins, 2018	30	29	-2.90 [-3.82; -1.98]	7.2%	19.2%
Ramchandani, 2021	80	84	-1.30 [-1.70; -0.90]	38.3%	25.6%
Tal, 2017	24	24	-2.00 [-3.35; -0.65]	3.4%	14.1%
**Common effect model**	**194**	**188**	**-1.39 [-1.64; -1.14]**	**100.0%**	**--**
**Random effects model**			**-1.84 [-2.56; -1.11]**	**--**	**100.0%**

Heterogeneity: $I^2 = 76\%$, $\tau^2 = 0.4924$, $p < 0.01$
Test for overall effect (common effect): $z = -11.03$ ($p < 0.01$)
Test for overall effect (random effects): $z = -4.97$ ($p < 0.01$)

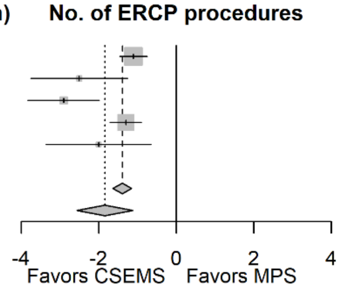

Fig. 21 Pooled estimate of number of ERCP - subgroup analysis based on etiology

Study	Total (CSEMS)	Total (MPS)	MD [95%-CI]	Weight (common)	Weight (random)
**Orthotopic Liver Transplant**					
Coté, 2016	33	31	-0.92 [-1.27; -0.57]	77.5%	35.9%
Kaffes, 2014	10	10	-2.50 [-3.75; -1.25]	6.1%	20.0%
Martins, 2018	30	29	-2.90 [-3.82; -1.98]	11.2%	25.6%
Tal, 2017	24	24	-2.00 [-3.35; -0.65]	5.2%	18.5%
**Common effect model**	**97**	**94**	**-1.29 [-1.60; -0.99]**	**100.0%**	**--**
**Random effects model**			**-1.99 [-2.97; -1.01]**	**--**	**100.0%**
Heterogeneity: $I^2 = 86\%$, $\tau^2 = 0.7475$, $p < 0.01$					
Test for effect in subgroup (common effect): $z = -8.23$ ($p < 0.01$)					
Test for effect in subgroup (random effects): $z = -3.99$ ($p < 0.01$)					
**Chronic Pancreatitis**					
Coté, 2016	15	8	-1.50 [-2.25; -0.75]	22.1%	45.1%
Ramchandani, 2021	80	84	-1.30 [-1.70; -0.90]	77.9%	54.9%
**Common effect model**	**95**	**92**	**-1.34 [-1.70; -0.99]**	**100.0%**	**--**
**Random effects model**			**-1.34 [-1.70; -0.99]**	**--**	**100.0%**
Heterogeneity: $I^2 = 0\%$, $\tau^2 = 0$, $p = 0.64$					
Test for effect in subgroup (common effect): $z = -7.47$ ($p < 0.01$)					
Test for effect in subgroup (random effects): $z = -7.47$ ($p < 0.01$)					

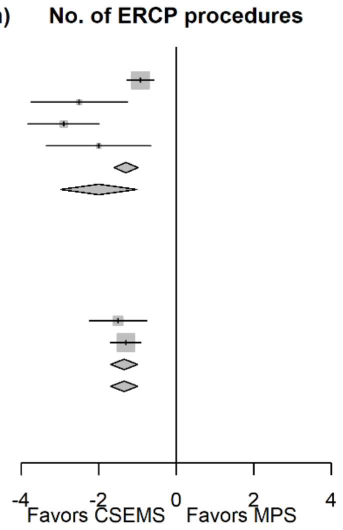

Test for subgroup differences (common effect): $\chi^2_1 = 0.04$, df = 1 ($p = 0.83$)
Test for subgroup differences (random effects): $\chi^2_1 = 1.50$, df = 1 ($p = 0.22$)

Fig. 22 Trial sequential analysis of number of ERCP

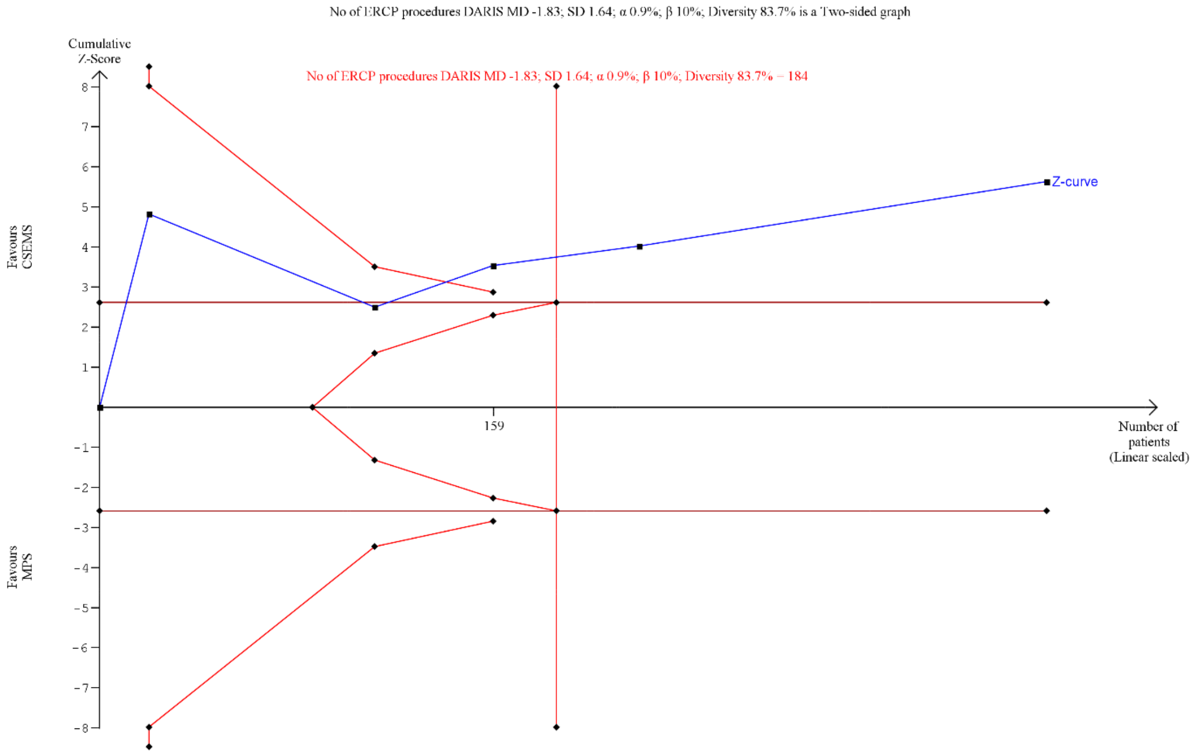

Fig. 23 Pooled estimate of number of stents – subgroup analysis based on etiology

Study	Total (CSEMS)	Total (MPS)	MD [95%-CI]	Weight (common)	Weight (random)
Postsurgical strictures (cholecystectomies)					
Artifon, 2012	15	16	-3.80 [-6.73; -0.87]	7.1%	14.2%
Mixed					
Coté, 2016	57	55	-0.82 [-2.32; 0.68]	27.2%	15.3%
Chronic Pancreatitis					
Haapamäki, 2015	28	30	-4.67 [-7.62; -1.72]	7.0%	14.2%
Ramchandani, 2021	80	84	-4.40 [-5.59; -3.21]	43.1%	15.5%
**Common effect model**	**108**	**114**	**-4.44 [-5.54; -3.33]**	**50.2%**	**--**
**Random effects model**			**-4.44 [-5.54; -3.33]**	**--**	**29.7%**
Heterogeneity: $I^2 = 0\%$, $\tau^2 = 0$, $p = 0.87$					
Test for effect in subgroup (common effect): $z = -7.88$ ($p < 0.01$)					
Test for effect in subgroup (random effects): $z = -7.88$ ($p < 0.01$)					
Orthotopic Liver Transplant					
Kaffes, 2014	10	10	-6.50 [-10.39; -2.61]	4.0%	13.2%
Martins, 2018	30	29	-15.70 [-18.60; -12.80]	7.3%	14.2%
Tal, 2017	24	24	-7.00 [-10.78; -3.22]	4.3%	13.3%
**Common effect model**	**64**	**63**	**-10.93 [-12.91; -8.95]**	**15.6%**	**--**
**Random effects model**			**-9.85 [-15.82; -3.89]**	**--**	**40.8%**
Heterogeneity: $I^2 = 90\%$, $\tau^2 = 24.5372$, $p < 0.01$					
Test for effect in subgroup (common effect): $z = -10.81$ ($p < 0.01$)					
Test for effect in subgroup (random effects): $z = -3.24$ ($p < 0.01$)					
**Common effect model**	**244**	**248**	**-4.42 [-5.20; -3.64]**	**100.0%**	**--**
**Random effects model**			**-6.04 [-9.55; -2.52]**	**--**	**100.0%**

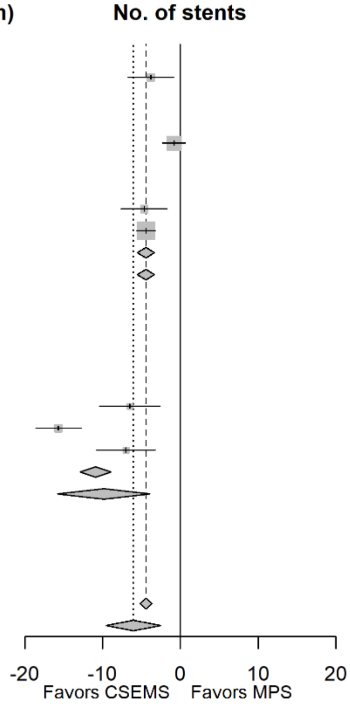

Heterogeneity: $I^2 = 93\%$, $\tau^2 = 20.4005$, $p < 0.01$
Test for overall effect (common effect): $z = -11.09$ ($p < 0.01$)
Test for overall effect (random effects): $z = -3.37$ ($p < 0.01$)
Test for subgroup differences (common effect): $\chi^2_3 = 63.78$, df = 3 ($p < 0.01$)
Test for subgroup differences (random effects): $\chi^2_3 = 19.32$, df = 3 ($p < 0.01$)

Fig. 24 Trial sequential analysis of number of stents

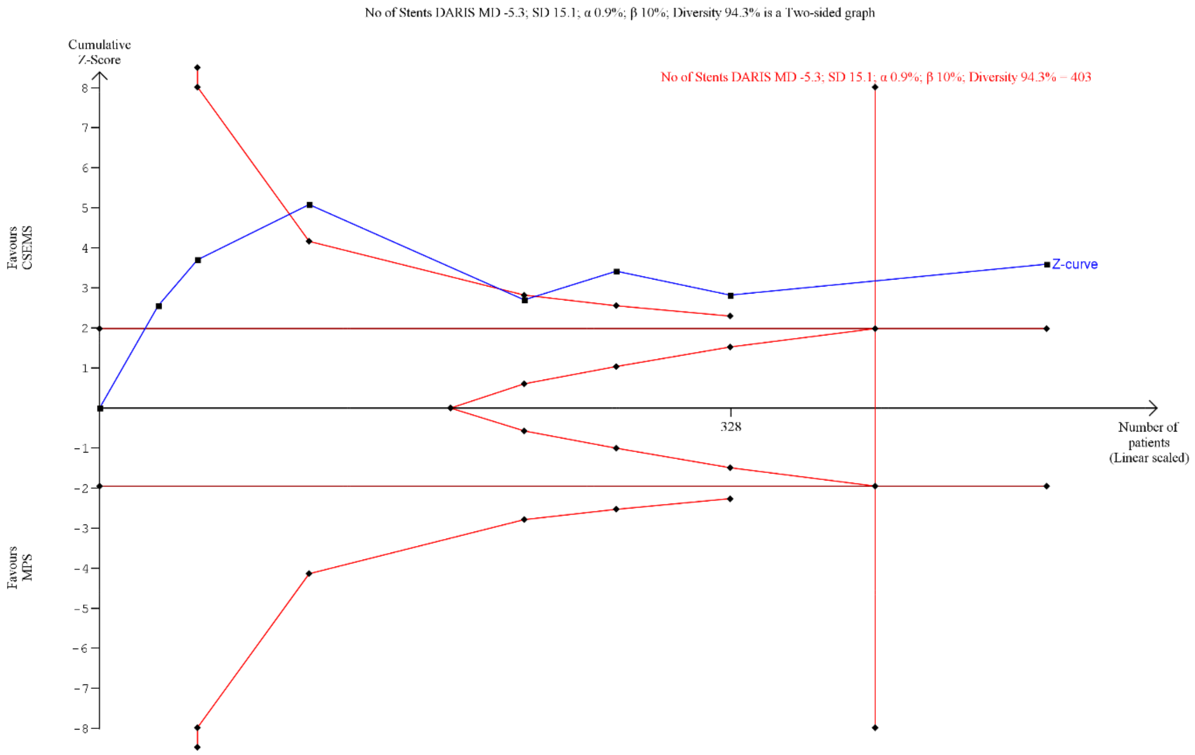

Fig. 25 Pooled estimate of number of stent treatment duration

Study	Total (CSEMS)	Total (MPS)	MD [95%-CI]	Weight (common)	Weight (random)
Coté, 2016	50	41	-44.00 [-91.82; 3.82]	14.8%	19.9%
Kaffes, 2014	10	10	-189.00 [-232.45; -145.55]	17.9%	20.1%
Martins, 2018	30	29	-203.40 [-233.78; -173.02]	36.7%	20.7%
Ramchandani, 2021	80	84	0.00 [-38.67; 38.67]	22.6%	20.4%
Tal, 2017	24	24	-51.00 [-115.97; 13.97]	8.0%	18.8%
**Common effect model**	**194**	**188**	**-119.02 [-137.41; -100.62]**	**100.0%**	**--**
**Random effects model**			**-98.62 [-180.80; -16.43]**	**--**	**100.0%**

Stent treatment duration (days)

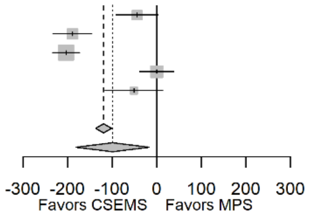

Heterogeneity: $I^2 = 96\%$, $\tau^2 = 8237.8277$, $p < 0.01$
Test for overall effect (common effect): $z = -12.68$ ($p < 0.01$)
Test for overall effect (random effects): $z = -2.35$ ($p = 0.02$)

Fig. 26 Pooled estimate of stent treatment duration – subgroup analysis based on etiology

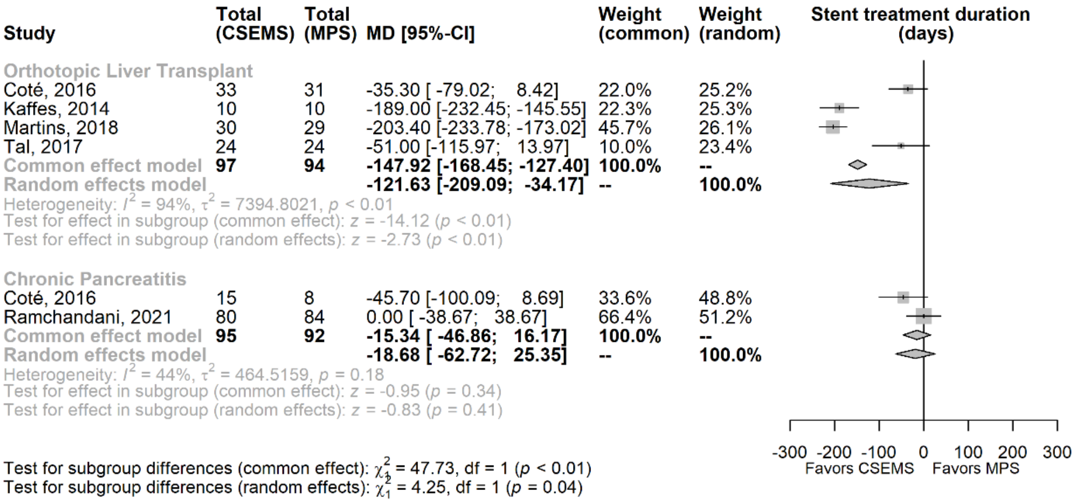

Fig. 27 Trial sequential analysis of stent treatment duration

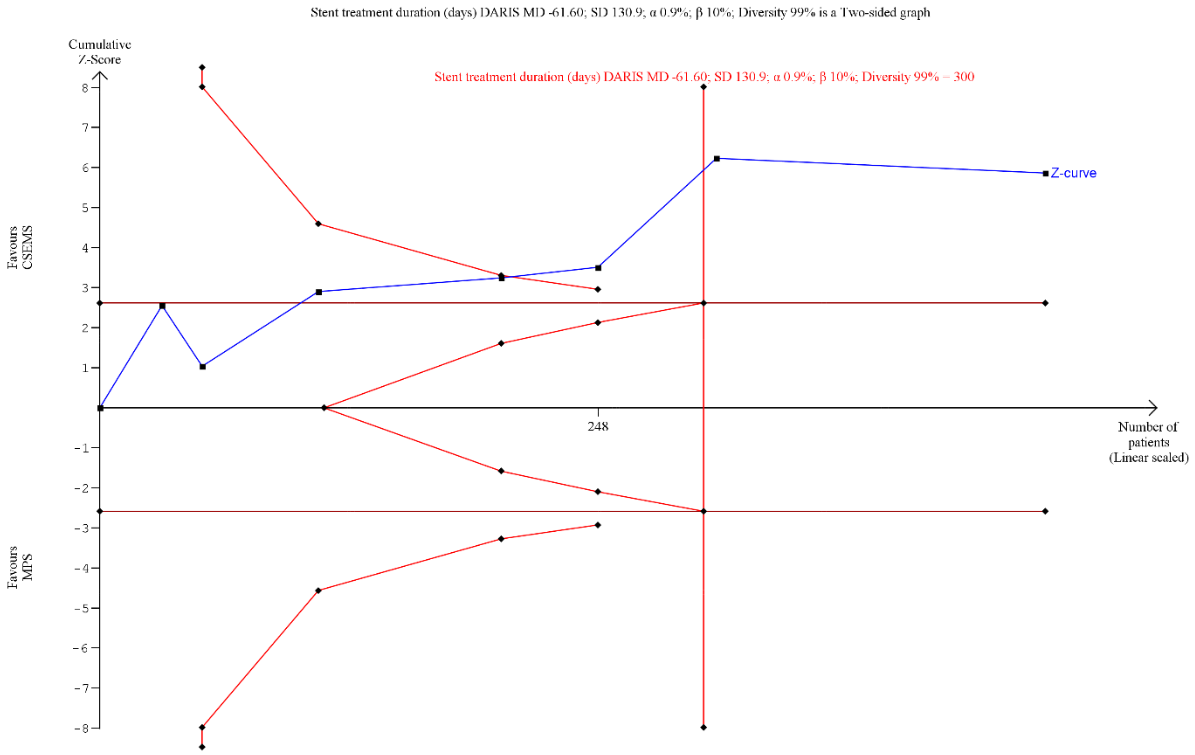

Supplement: Supplementary file 1 — Supplementary Material [file 10-1055-s-0046-1818623-s250066.pdf]
